# Expression profile and prognostic values of LSM family in skin cutaneous melanoma

**DOI:** 10.1186/s12920-022-01395-6

**Published:** 2022-11-12

**Authors:** Xiaofang Sun, Jianping Zhang, Can Xiao, Zili Ge

**Affiliations:** 1 Department of Oral and Maxillofacial Surgery, The First Affiliated Hospital of Soochow University, Soochow University, Jiangsu, China; 2 Department of Dermatology, The Second Hospital of Jiaxing Affiliated to Jiaxing University, Jiaxing, Zhejiang China

**Keywords:** Biomarker, Skin cutaneous melanoma (SKCM), LSM family, LSM2, RNA splicing

## Abstract

**Background:**

The like-Smith (LSM) family is a group of RNA-binding proteins involved in RNA metabolism. However, their involvement in tumors, particularly skin cutaneous melanoma (SKCM), is not fully understood. In this study, we focused on the expression profiles and prognostic values of the LSM family in SKCM.

**Methods:**

Raw data were downloaded from The Cancer Genome Atlas. The expression profile and prognostic value of LSM genes in SKCM were explored using the GEPIA, cBioPortal, and HPA databases. Protein–protein and gene–gene interaction analyses were performed using STRING and GeneMANIA. Enrichment and Cox regression analysis were conducted using R software. The TISIDB database was used to explore the relationship between LSMs and immunomodulators. Receiver operating characteristic curves and nomogram models were constructed to validate prognostic values.

**Results:**

mRNA and protein expression levels of LSM2, LSM4, and LSM12 were significantly elevated in SKCM. The upregulated mRNA expression of LSM2 (*p* = 0.0013) and LSM4 (*p* = 0.0043) was significantly correlated with poor overall survival in patients with SKCM, whereas only LSM2 (*p* = 0.049) overexpression was markedly associated with worse disease-free survival. LSM2 overexpression was an independent risk factor (*p* = 0.013) and was confirmed to have a high prognostic value in SKCM using the receiver operating characteristic curve (AUC = 0.942) and nomogram models. All LSM genes were identified as genomic mutations, whereas alteration of LSM2 (*p* = 0.0153) significantly affected the overall survival in patients with SKCM. Significant correlations were observed between LSM family expression, immune cell infiltration, and immunomodulator. Furthermore, function and pathway enrichment analysis showed that the LSM family was mainly RNA binding proteins and involved in RNA splicing and degradation.

**Conclusion:**

Expression profiles and prognostic values of LSM in SKCM were inconsistent. Among the LSM family, only LSM2 may serve as a potential poor prognosticator and immunotherapeutic target of SKCM.

**Supplementary Information:**

The online version contains supplementary material available at 10.1186/s12920-022-01395-6.

## Background

Although skin cutaneous melanoma (SKCM) constitutes less than 5% of all skin cancers, it accounts for more than 75% of skin cancer related deaths [1]. The incidence and morbidity of SKCM have been steadily increasing worldwide over the past few decades, with 350,000 new cases and 60,000 deaths worldwide annually [2, 3]. The incidence of SKCM varies among ethnic groups, with the White population having the highest incidence (approximately 21.6/100,000 people), followed by the Black population (approximately 1.0/100,000 people), and the incidence rate in the Chinese population is slightly lower than that in the Black population [4, 5]. In recent years, the incidence of SKCM in China has increased significantly, with approximately 20,000 new cases occurring every year [6]. Therefore, early diagnosis and treatment are imperative to decrease SKCM-related mortality. Despite the development of surgical and systemic therapies [7], the survival rate of patients with SKCM remains relatively low. In terms of precision medicine, various studies have revealed that differentially expressed genes (DEGs) could be helpful in clarifying the tumor molecular biology, prognosis, and molecular targeted drugs in SKCM [8, 9]. To date, a large number of biomarkers have been discovered [10–12], but most are in the basic research stage, and there are only a few biomarkers that can be used to guide clinical practice. Therefore, identifying highly specific and selective biomarkers associated with SKCM is particularly important, which may facilitate the diagnosis, treatment, and prognosis of SKCM in the future.

Sm protein was first studied in research on precursor RNA. It is a highly conserved homologous protein family containing Sm motifs; therefore, it is called the Like-Smith (LSM) protein [13]. The conserved folding of Sm and LSM subunits consist of a 5-strand antiparallel β sheet, and in some cases it can be enhanced by the addition of secondary structural elements and/or unstructured C-terminal extensions of varying length [14]. There are 13 members of the LSM protein family (LSM1-LSM14B). The LSM protein family is an RNA binding protein (RBP) that is mainly involved in the shearing and processing of precursor mRNA in the nucleus [13, 15]. In eukaryotic cells, a hetero-heptameric complex of seven LSM proteins (LSM1-7) promotes mRNA decapping and decay in the cytoplasm, whereas a different hetero-heptameric complex of LSM proteins (LSM2-8) affects the processing of pre-mRNA and small stable RNAs in the nucleus [16, 17]. The LSM1-7 complex physically interacts with other decay factors, such as Pat1p, Dhh1p, and Xrn1p [18] and preferentially associates in vivo with a pool of oligoadenylated mRNPs targeted for decapping enzyme rather than the translation factors [19, 20]. The LSM2-8 complex shares six out of seven subunits with the LSM1-7 complex, and binds the 3′ ends of U6 and U6^atac^ snRNAs. The U6 snRNA is then processed by the 3′-5′ exoribonuclease Usb1, resulting in a 2′, 3′ cyclic phosphate in most organisms [21]. LSM12 was identified as an essential participant in NAADP-evoked TPC activation and Ca^2+^ mobilization from acidic stores [22]. The oncologic roles of the LSM family have been explored in several cancers. LSM1 participates in the proliferation of viruses in cells and regulates their replication [23, 24]; it also acts as an oncogene in breast cancer (BC) after 8p11-12 amplification [25]. Moreover, LSM1 promotes chemoresistance, transformation, and metastasis of pancreatic cancer cells [26]. Meanwhile, downregulation of LSM1 is associated with prostate cancer progression [27]. LSM2 homologous U6 small nuclear RNA-associated form, namely the LSM2-8 complex, participates in the splicing of mRNA precursors and regulates the expression of multiple genes by controlling the spliceosomal cycle. RNA processing and stabilization are also maintained in the nucleus [17]. In ovarian cancer, the decrease in the degree of LSM2 methylation may lead to an increase in its expression, which results in a disorder in the processing and circulation of most RNA, affecting the expression of various proteins and accelerating tumor development [28]. LSM3 has been confirmed to be not only major pathogenic gene of Alzheimer’s disease (AD), but also bridge gene [29] and to significantly affect the proliferation of non-small cell lung cancer cell lines [30]. In addition, LSM4 overexpression was significantly correlated with poor survival in BC [31], pancreatic ductal adenocarcinoma (PDAC) [32], and hepatocellular carcinoma (HCC) [33]. Overall, these studies indicate that the LSM family may be a promising therapeutic target and prognostic biomarker for multiple tumors. However, the functions of LSM family members have not been fully elucidated. In particular, there are no reports about LSM family members in SKCM.

With the continuous progress of bioinformatics and bioinformatic approaches, comprehensive analysis of gene expression, epigenetic regulation, and biological functions in numerous malignancies is becoming increasingly accessible. Hence, we assessed the expression patterns, prognostic values, and biological functions of LSM family genes in SKCM using multiple public databases. We found that the expression profiles and prognostic values of LSM family members in SKCM were inconsistent. Only LSM2, in the LSM family, might serve as a potential biomarker for SKCM.

## Methods

### The cancer genome atlas

The Cancer Genome Atlas (TCGA) is a large tumor gene chip database that can be used to identify outliers, analyze differences in gene expression, and predict co-expressed genes. The clinical data in TCGA includes tumor stage, tissue type, grade, and overall survival. It can be used to identify DEGs, therapeutic targets, and diagnostic biomarkers. The RNA sequencing data and clinical data of SKCM were downloaded from the Genomic Data Commons (GDC, https://portal.gdc.cancer.gov/).

### Gene expression profiling interactive analysis

Gene Expression Profiling Interactive Analysis (GEPIA; http://gepia.cancer-pku.cn/) is an online database. It is a network assistant in exploring the RNA sequencing expression of 9736 tumors and 8587 regular cases from TCGA and Genotype Tissue Expression (GTEx) by applying a qualified processing method [34]. In this study, GEPIA was used to explore LSM family gene expression profiles and survival analysis.

### Human protein atlas

The Human Protein Atlas (HPA; https://www.proteinatlas.org/) database was used to determine the protein levels of the LSM family in SKCM and normal skin tissues.

### cBiPortal

The cBio Cancer Genomics Portal (cBioPortal, https://www.cbioportal.org/) is a platform based on TCGA that provides multidimensional visual data. A total of 2549 cutaneous melanoma samples were used for further analysis. The type and frequency of LSM family gene mutations in cutaneous melanoma were analyzed using the “OncoPrint” and “Cancer Type Summary” module. The prognostic value of gene alterations was explored in the “survival” module.

### GeneMANIA analysis

GeneMANIA (http://genemania.org/) is a user-friendly tool. It can predict the interactions between genes, including co-expression, co-locations, pathways, physical interactions, and genetic interactions, to generate prioritized genes for further functional analysis.

### STRING

STRING (https://cn.string-db.org/) was used to probe known interactions between proteins and help speculate their interactions. In our study, STRING was used for protein–protein interaction (PPI) analysis of the LSM family and peripheral interaction proteins. Statistical significance was set at a confidence score > 0.4 was considered significant.

### Function enrichment analysis

Gene Ontology (GO), which includes three categories: biological process (BP), cellular component (CC), and molecular function (MF), can provide a framework to reveal the function of gene products and to clarify transcriptome data of high-throughput genes and biological properties. The Kyoto Encyclopedia of Genes and Genomes (KEGG) [35] can identify specific pathways that can be used for the functional interpretation of genomic information. GO terms and KEGG pathways enrichment analysis were performed by “clusterProfiler” R package. An adjusted *p*-value < 0.05 was considered as significant enrichment.

### Immune-related analysis

First, the R package “GSVA” was used to present infiltration enrichment of 24 common immune cells, including T helper cells, type 2 Th (Th2) cells, CD8 T cells, T central memory (Tcm), Th1 cells, T gamma delta (Tgd), B cells, T cells, dendritic cell (DC), activated dendric cells (aDC), plasmacytoid DC (pDC), immature DC (iDC), natural killer (NK) cells, NK CD56bright cells, Cytotoxic cells, type 17 Th (Th17) cells, T follicular helper (TFH), NK CD56dim cells, regulatory T cell (Treg), macrophages, mast cells, neutrophils, Tem, and eosinophils. The relationship between LSM gene expression and immune cell infiltration levels was evaluated using Spearman’s analysis.

TISIDB (http://cis.hku.hk/TISIDB/index.php) is a user-friendly web portal for the analysis of interactions between tumors and immunomodulatory factors. It integrates multiple heterogeneous data types, including RNA sequencing data of patients receiving immunotherapy, literature mining results from PubMed, high-throughput screening data, and TCGA data [36]. Here, to further clarify the immune correlation of LSMs in SKCM, we used the “Immunomodulator” module in the TISIDB database to explore and evaluate the relationship between LSM expression and the level of immune checkpoint genes.

### Statistical analysis

The receiver operating characteristic (ROC) curve analysis according to sensitivity and specificity was performed using the “pROC” and “ggplot2” packages. (R software, version 4.1.3, https://www.r-project.org/). The area under the curve (AUC) ranging from 0.5 (no diagnosis) to 1.0 (perfect diagnosis) was established. The interactions of LSM family genes, as well as the relationship between LSM family gene expression and immune cell infiltration, were determined using Spearman’s analysis. The univariate and multivariate Cox regression analysis were used to analyze the individual hazard ratio (HR) for overall survival (OS) via the “survival” R package. HR with a 95% confidence interval (CI) was measured to estimate the hazard risk of individual factors. The “rms” R package was used to draw the nomogram and construct a prediction model. A *p*-value < 0.05 was considered significantly different.

## Results

### Aberrant expression of LSM family genes in patients with SKCM

**Fig. 1:**
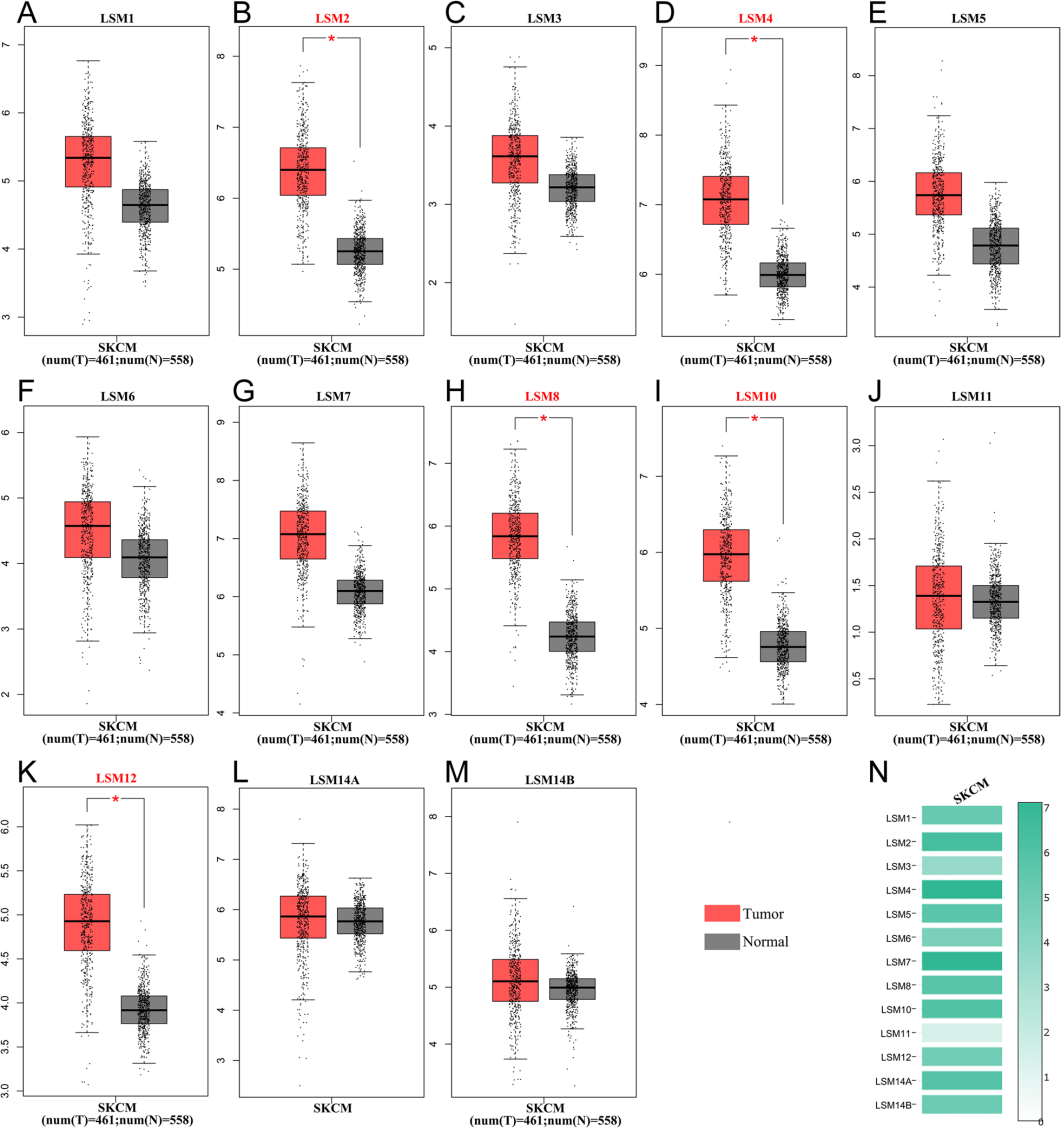
Differential expression of LSM family genes between the SKCM samples and normal skin tissues at mRNA level. LSM2, LSM4, LSM8, LSM10, and LSM12 were significantly overexpressed in SKCM (n = 461) compared with normal skin tissues (n = 558). Red box, tumor; grey box, normal samples. T, tumor; N, normal. *p < 0.05. N Expression profile of LSM family members in SKCM samples. Abbreviations: LSM, Like-Smith; SKCM, skin cutaneous melanoma

**Fig. 2:**
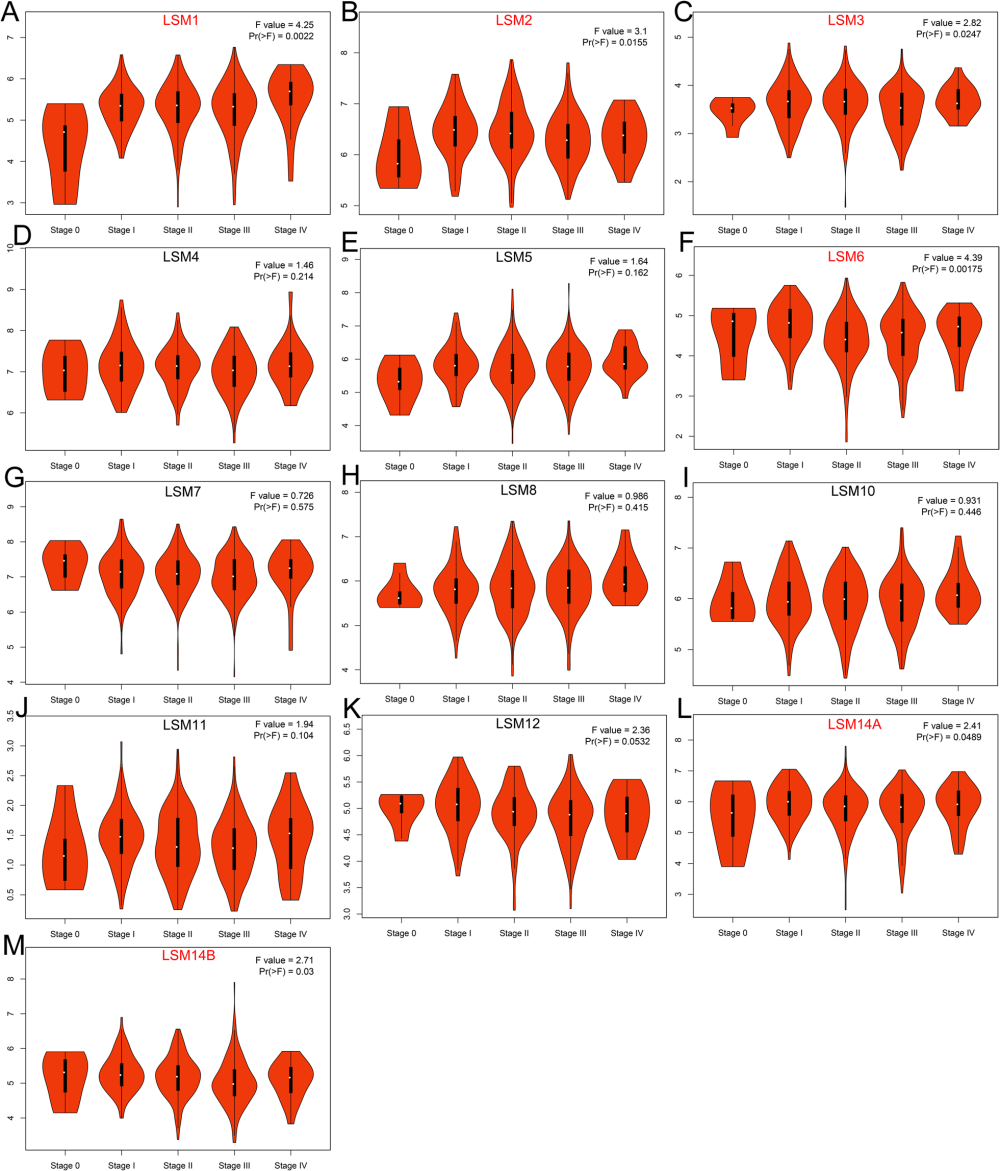
Associations between the expression of LSM genes and the pathological stage in patients with SKCM. The expression of LSM1, LSM2, LSM3, LSM6, LSM14A, and LSM14B was significantly correlated with the tumor stage, *p* < 0.05

**Fig. 3:**
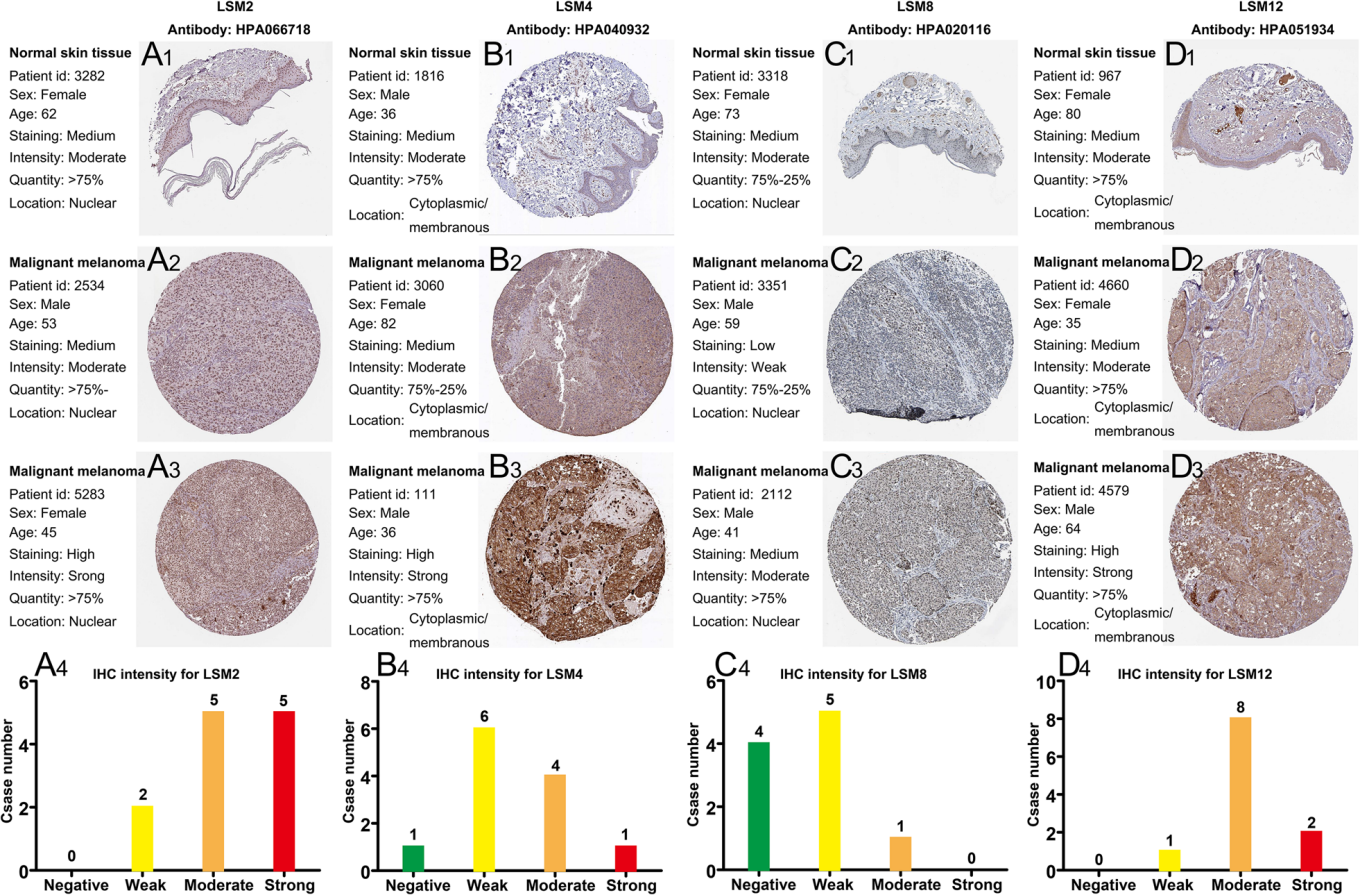
The IHC images of upregulated LSM family members (LSM2, LSM4, LSM8, and LSM12) in SKCM patients. **A**_**1**_, **B**_**1**_, **C**_**1**_ and **D**_**1**_ represent the protein levels of LSM2, LSM4, LSM8, and LSM12, respectively, in normal skin tissue. **A**_**2–3**_, **B**_**2–3**_, **C**_**2–3**_, and **D**_**2–3**_ represent the protein expression of LSM2, LSM4, LSM8, and LSM12 in cutaneous melanoma tissues. The IHC staining intensities of LSM2 (12 patients), LSM4 (12 patients), LSM8 (10 patients), and LSM12 (11 patients) are shown in the bar charts (**A**_**4**_, **B**_**4**_, **C**_**4**_, and **D**_**4**_). All data were obtained from HPA. Abbreviations: IHC, immunohistochemistry; HPA, human protein albumin

Based on the GEPIA database, the expression levels of LSM family members were compared between 461 SKCM tissues and 558 normal skin tissues. The expression levels of LSM2, LSM4, LSM8, LSM10, and LSM12 were remarkably elevated in SKCM specimens compared to normal cutaneous specimens (Fig. 1B, D, H, I, K). There were no statistically significant differences in the expression of LSM1, LSM3, LSM5, LSM6, LSM7, LSM11, LSM14A, and LSM14B compared with that in normal tissue (Fig. 1A, C, E, F, G, J, L, M). A summary of LSM family expression in SKCM is shown in Fig. 1N. We further assessed the LSM family expression at various pathological stages (Fig. 2). The results showed that the expression of LSM1, LSM2, LSM3, LSM6, LSM14A, and LSM14B (Fig. 2A–C, F, L, M) were significantly correlated with the pathological stages (*p* < 0.05). The expression of LSM4, LSM5, LSM7, LSM8, LSM10, LSM11 and LSM12 were not related to the pathological stages (Fig. 2 D, E, G, H, I, J, K). Subsequently, the protein expression patterns of LSM genes in patients with SKCM were explored using the HPA database. Immunohistochemical images of LSM2 (Fig. 3A1–A4), LSM4 (Fig. 3B1–B4), LSM8 (Fig. 3C1–C4), and LSM12 (Fig. 3D1–D4) in patients with SKCM are presented in Fig. 3. LSM8 was weakly expressed in cutaneous melanoma and normal tissues. The protein expression of LSM2, LSM4, and LSM12 was upregulated in cutaneous melanoma. Most of the LSM2 cells were strongly stained in melanoma tissues compared to the normal control.

### Prognostic values of LSM family in SKCM

**Fig. 4:**
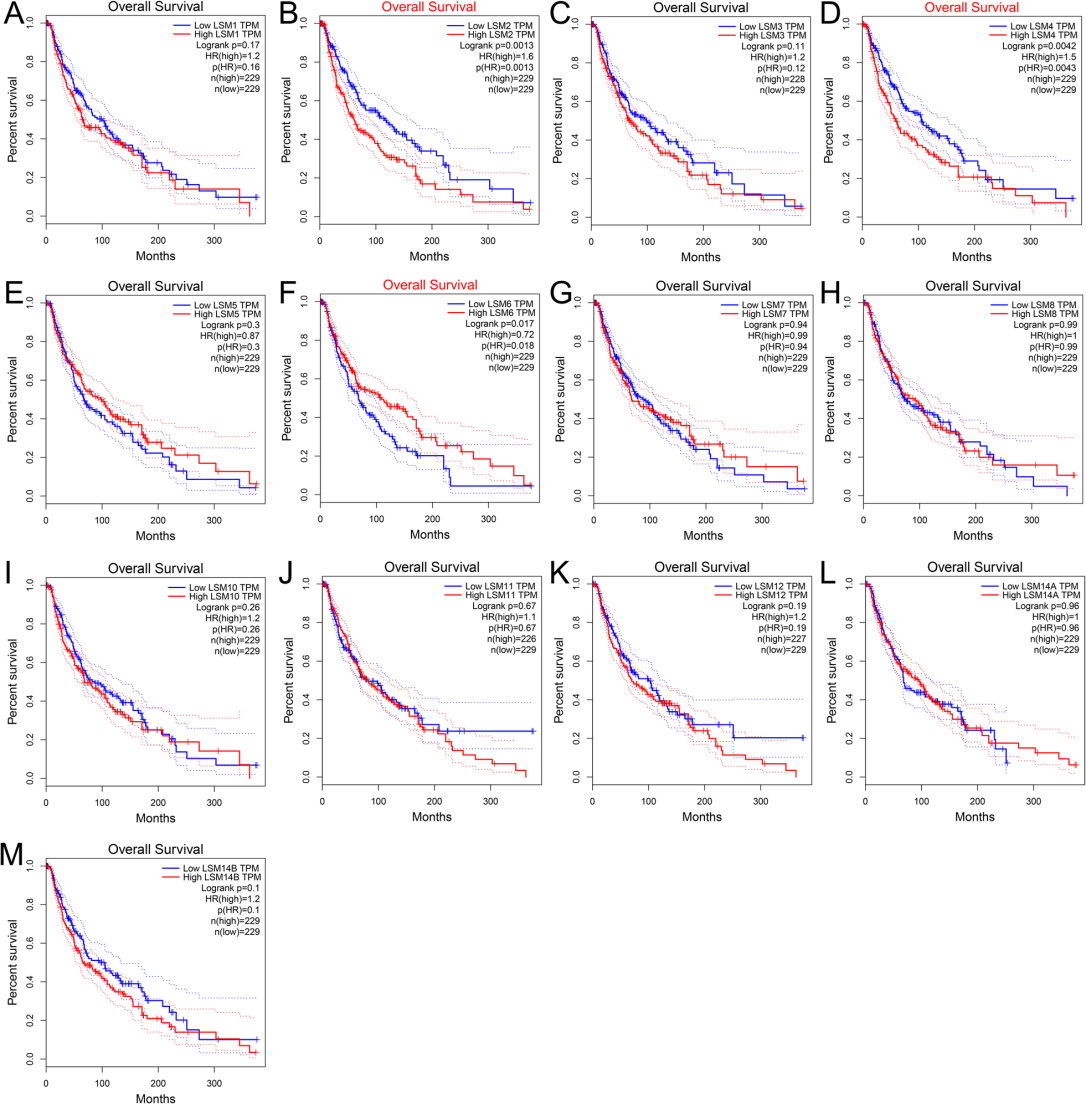
The relationships between OS and the expression of LSM family members in SKCM patients. Overexpression of LSM2 (*p* = 0.0013) and LSM4 (*p* = 0.0043) was associated with poor OS in SKCM. Upregulated expression of LSM6 (*p* = 0.018) was associated with longer OS in patients with SKCM. Abbreviations: OS, overall survival

**Fig. 5:**
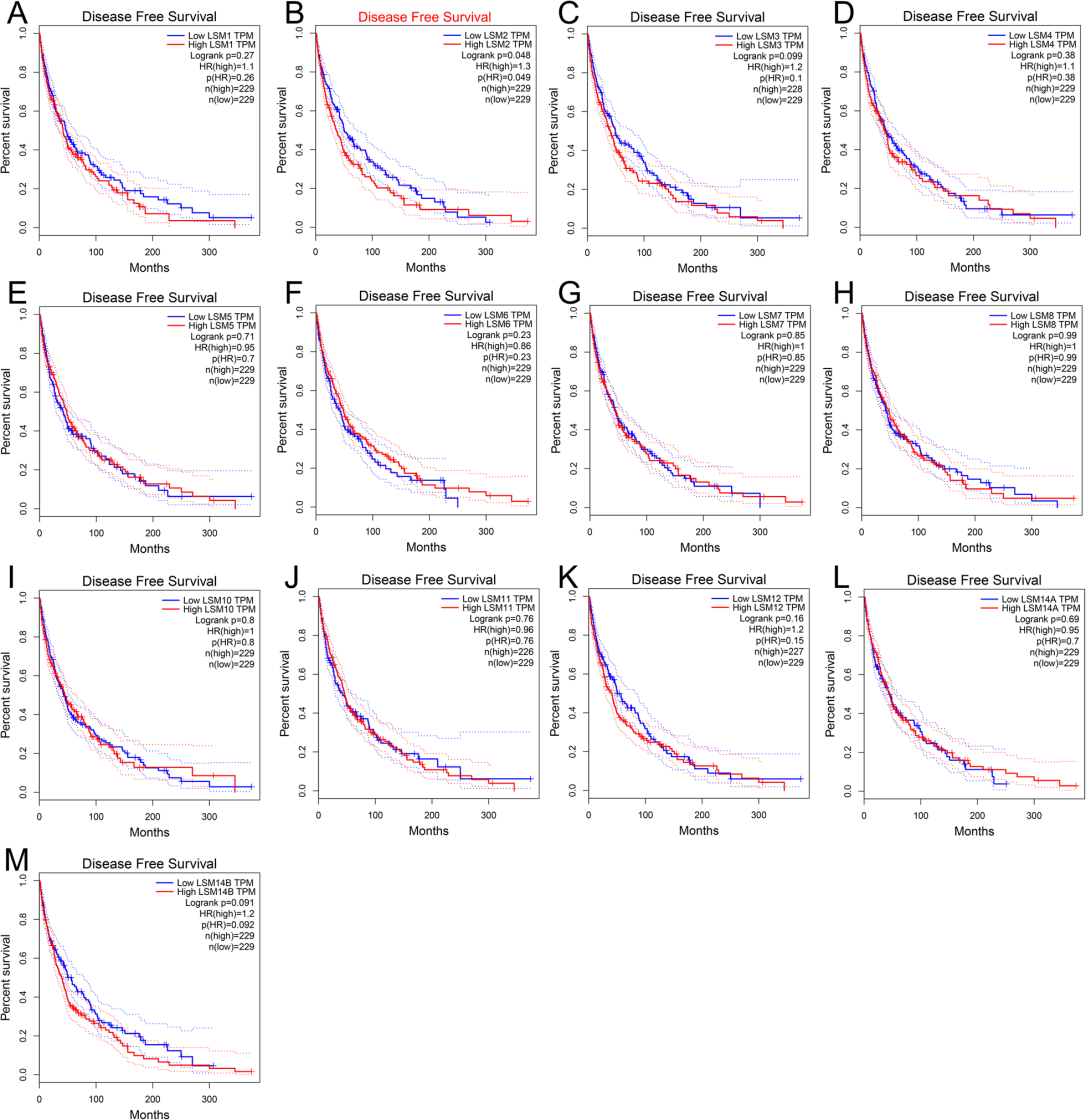
The correlations between the DFS and the expression of LSM genes in patients with SKCM. In particular, LSM2 overexpression significantly affected the DFS of SKCM patients (*p* = 0.049). Abbreviations: DFS, disease-free survival

**Fig. 6:**
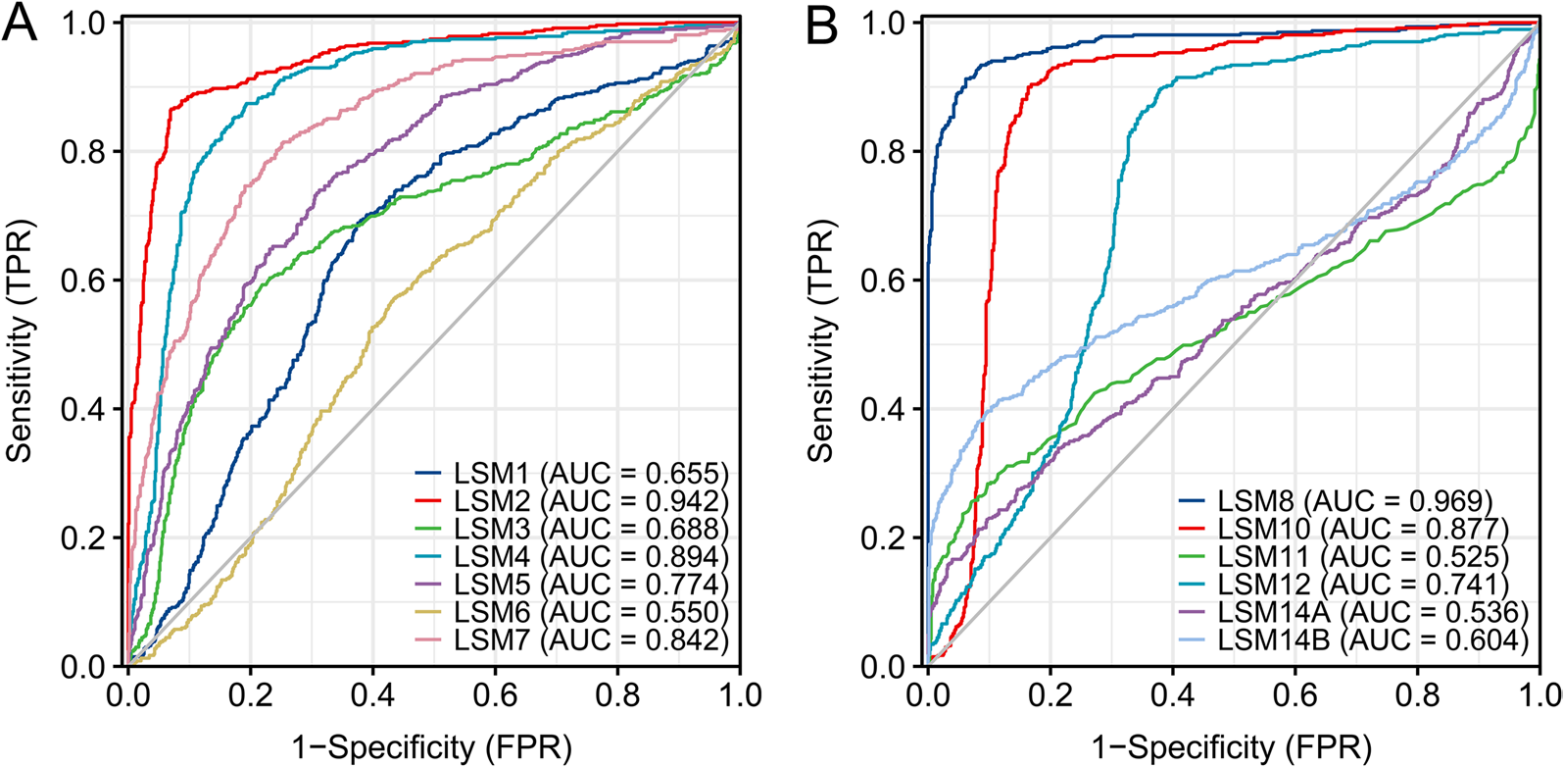
Assessment of prognostic values of LSM genes by ROC curves. **A** The ROC curve of LSM1-LSM7. **B** ROC curve of LSM8-LSM14. The results indicated that LSM2, LSM4, LSM7, LSM8, and LSM10 had high accuracy (AUC > 0.8), whereas LSM1, LSM3, LSM5, LSM6, LSM9, LSM11, LSM12, LSM14A, and LSM14B had low accuracy in predicting SKCM. Abbreviations: ROC, receiver operating characteristic; AUC, area under the curve

The GEPIA database was used to investigate the prognostic value of LSM family members in SKCM. The results suggest that LSM2, LSM4, and LSM6 were significantly correlated with OS. Higher expression of LSM2 (*p* = 0.0013) and LSM4 (*p* = 0.0043) and lower expression of LSM6 (*p* = 0.018) were prognostic factors for OS (Fig. 4B, D, F). The other LSMs had no significant influence on OS (Fig. 4 A, C, E, G, H, I, J, K, L, M). However, only LSM2 (Fig. 5B) was closely linked to disease-free survival (DFS), and patients with low LSM2 expression had better DFS (*p* = 0.049). LSM1, LSM3, LSM4, LSM5, LSM6, LSM7, LSM8, LSM10, LSM11, LSM12, LSM14A, and LSM14B expressions were not associated with DFS (Fig. 5A, C, D, E, F, H, I, J, K, L, M). In univariate analysis, the HR of LSM2, LSM4, and LSM6 were 1.354, 1.354, and 0.593, respectively, which were significantly related to patient survival (Additional file 1: Figure S1). Some members of the LSM family were significantly upregulated, and their overexpression was associated with several clinical characteristics and adverse clinical outcomes in patients with SKCM. We further evaluated the prognostic value of LSM genes in patients with SKCM. ROC curve analysis showed that LSM2 (AUC = 0.942), LSM4 (AUC = 0.894), LSM7 (AUC = 0.842), LSM8 (AUC = 0.969), and LSM10 (AUC = 0.877) had high accuracy, whereas LSM1 (AUC = 0.655), LSM3 (AUC = 0.688), LSM5 (AUC = 0.774), LSM6 (AUC = 0.550), LSM9 (AUC = 0.942), LSM11 (AUC = 0.525), LSM12 (AUC = 0.741), LSM14A (AUC = 0.536), and LSM14B (AUC = 0.604) had low accuracy in predicting the prognosis of SKCM (Fig. 6).

### Gene mutation of LSM family genes

**Fig. 7:**
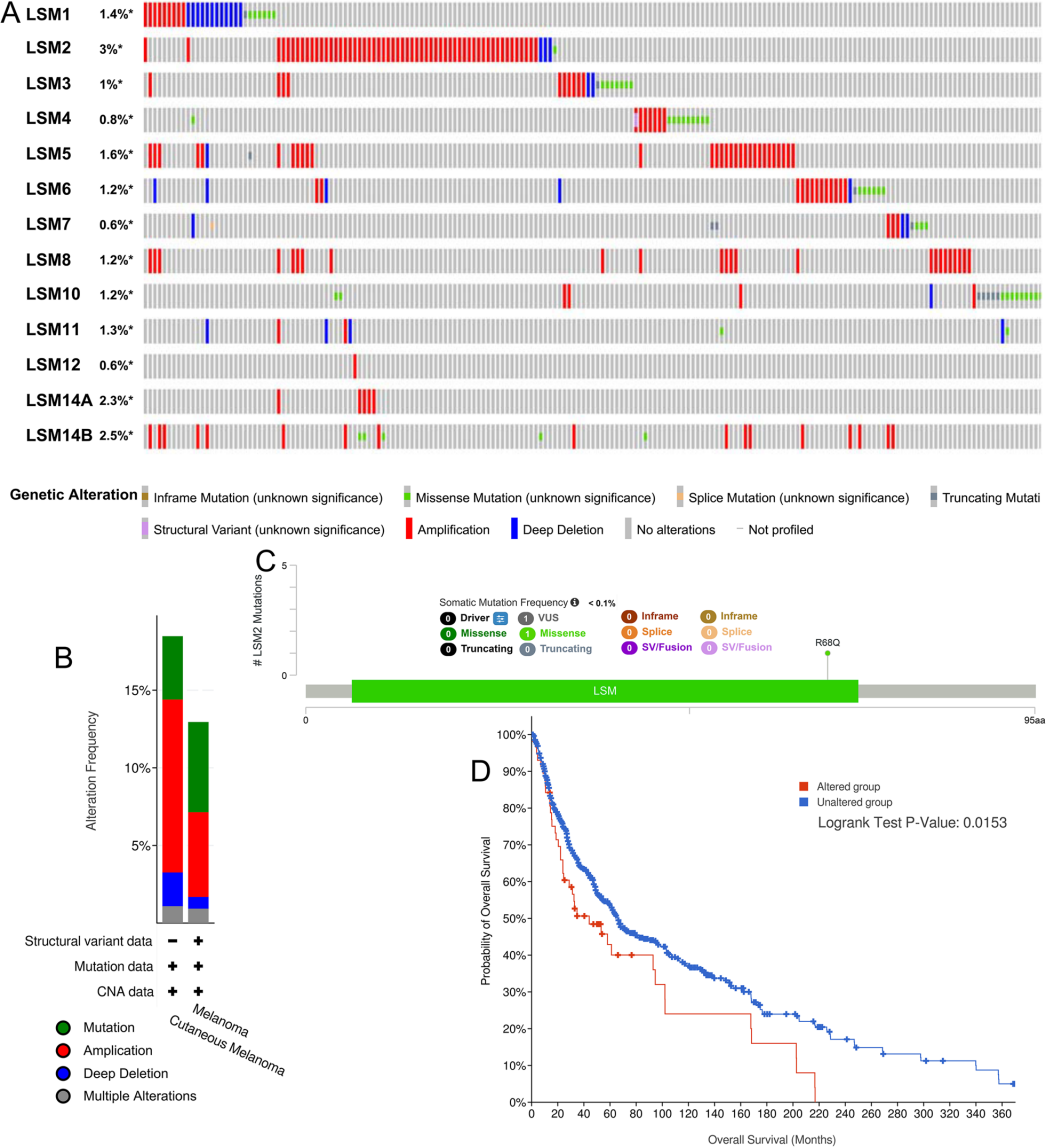
Alterations of LSM genes and the results obtained from survival analysis in SKCM. **A** OncoPrint visual summary of LSM gene alterations. **B** Summary of alterations in LSM family genes in cutaneous melanoma. **C** Mutation of LSM2 in SKCM. D. Kaplan–Meier plot comparing the OS in patients with and without LSM2 alterations in SKCM (*p* = 0.0153)

cBioPortal was used to explore the genetic alterations differentially expressed in LSM family genes. Our findings showed that LSM1 (1.2%), LSM2 (3%), LSM3 (1%), LSM4 (0.9%), LSM5 (1.3%), LSM6 (1.1%), LSM7 (0.6%), LSM8 (1.2%), LSM10 (1.3%), LSM11 (1.3%), LSM12 (0.6%), LSM14A (2.2%), and LSM14B (2.4%) were altered in SKCM samples (Fig. 7A). Of the 368 cases, 18.48% exhibited alterations in LSM family genes, including mutation (4.08%; 15 cases), amplification (11.14%; 41 cases), deep deletion (2.17%; 8 cases), multiple alterations (1.09%; 4 cases) (Fig. 7B). The mutation type of LSM2 was missense (Fig. 7C). The altered LSM2 (*p* = 0.0153) significantly affected the OS of patients with SKCM (Fig. 7D).

### Correlation and functional enrichment analysis of LSM family genes in SKCM patients

**Fig. 8:**
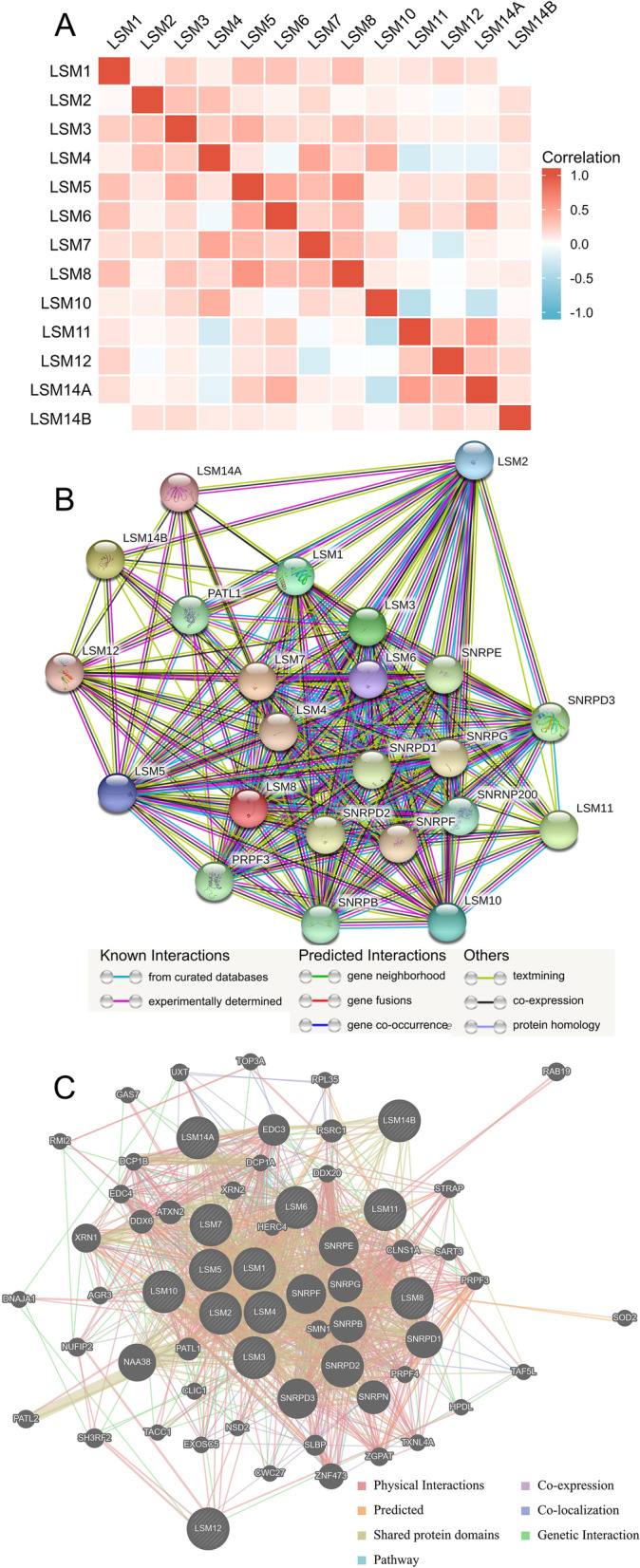
The correlations between LSM family and related genes. **A** The heatmap of LSM family gene correlations was analyzed using the Spearman test. **B** Protein–protein interaction network of LSM family genes. **C** Gene–gene interaction association networks of LSM family genes and the top 50 co-expression genes

**Fig. 9:**
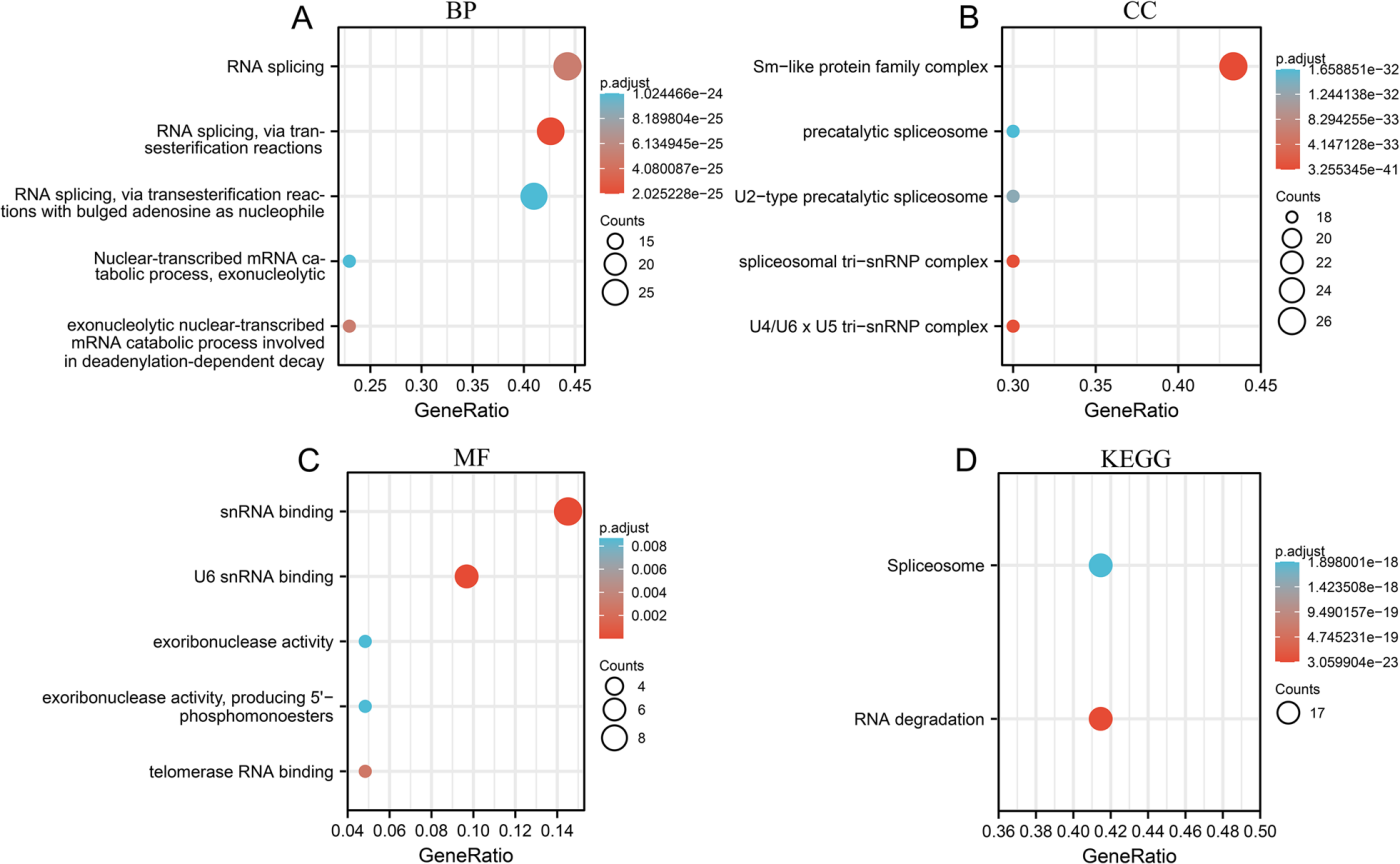
GO and KEGG analysis of the LSM family and top 50 involved genes in SKCM. **A**–**C** display the top five BP, CC, and MF, respectively. **D** KEGG pathways of the genes involved. Functional and pathway enrichment was determined using R and displayed in bubble charts. Abbreviations: GO, Gene Ontology; KEGG, Kyoto Encyclopedia of Genes and Genomes; BP, biological process; CC, cellular component; MF, molecular function

The correlation coefficients of the LSM family in SKCM were assessed and are shown in Fig. 8 A (The details of Spearman analysis and *p*-value are shown in Additional file 4: Tables S1, Additional file 5: Table S2). LSM5 expression was closely correlated with that of LSM10 (R = 0.57, *p* < 0.05). LSM8 expression closely correlated with LSM6 expression (R = 0.57, *p* < 0.05). LSM11 expression closely correlated with LSM14B expression (R = 0.53, *p* < 0.05). The PPI network revealed associations between the LSM family genes (Fig. 8B). Furthermore, a network of LSM family genes and functionally related genes was constructed using GeneMANIA. The top 50 most related genes of the LSM family is shown in Fig. 8C. SNRPD2, SNRPD3, SNRPF, and SNRPE were identified as the genes most related to the LSM family. Functional and pathway enrichment of the above genes was performed and displayed in bubble charts using the R software (adjusted *p* < 0.05, Fig. 9). The main BP (Fig. 9A) of these genes was RNA splicing. The CC (Fig. 9B) of these genes corresponds to the Sm-like protein family complex. The MF (Fig. 9C) of these genes were most closely related to small nuclear RNA (snRNA) binding and U6 snRNA binding. KEGG (Fig. 9D) pathway analysis revealed that these genes were mainly involved in spliceosome and RNA degradation.

### Associations of LSM family and immune cell infiltration in SKCM

**Fig. 10:**
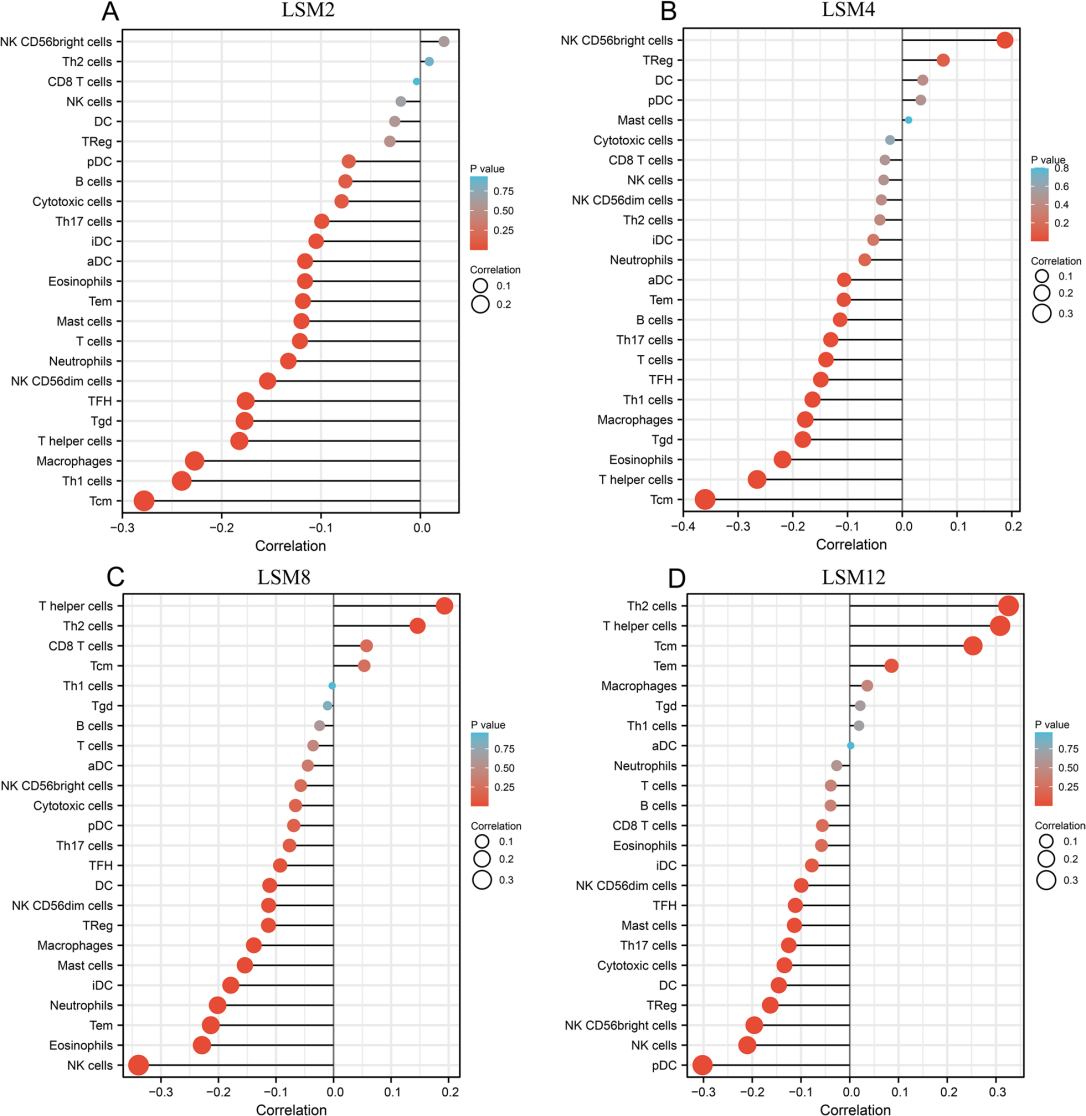
Correlations between immune infiltrating cells and the overexpression of LSM genes. **A** LSM2, **B** LSM4, **C** LSM8, and **D** LSM12, respectively

Tcm (R = −0.278, *p* < 0.001), Th1 cells (R = −0.24, *p* < 0.001), and macrophages (R = −0.227, *p* < 0.001) negatively correlated with LSM2 expression (Fig. 10A). Tcm (R = −0.36, *p* < 0.001) and T helper cells (R = −0.265, *p* < 0.001) were negatively correlated with LSM4, while NK CD56bright cells (R = 0.187, *p* < 0.001) were positively correlated (Fig. 10B). NK cells (R = -0.339, *p* < 0.001) and eosinophils (R = −0.229, *p* < 0.001) were negatively correlated with LSM8, while T helper cells (R = 0.193, *p* < 0.001) and Th2 cells (R = 0.146, *p* < 0.001) were positively correlated (Fig. 10C). NK cells (R = −0.21, *p* < 0.001) and pDC (R = −0.302, *p* < 0.001) were negatively correlated with LSM12, while Th2 cells (R = 0.326, *p* < 0.001) and T helper cells (R = 0.308, *p* < 0.001) were positively correlated (Fig. 10D).

### Regulation of immune molecules by LSM2 and LSM4

**Fig. 11:**
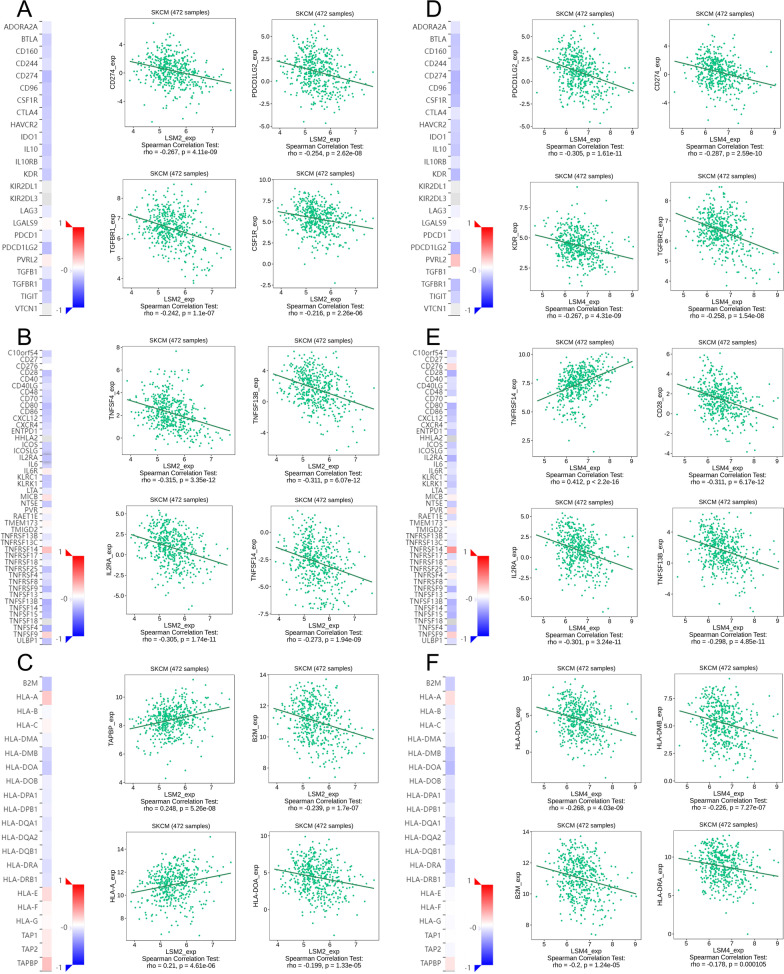
Spearman’s correlation of LSM2 and LSM4 expression with immunomodulators (TISIDB). **A**. Spearman correlation between Expression (exp) of LSM2 and Immunoinhibitors across SKCM. Top 4 immunoinhibitors displaying the greatest Spearman’s correlation with LSM2 expression. **B** Spearman correlation between Expression (exp) of LSM2 and Immunostimulators across SKCM. Top 4 immunostimulators displaying the greatest correlation with LSM2 expression. **C** Spearman correlation between Expression (exp) of LSM2 and MHC across SKCM. Top 4 MHC molecules displaying the greatest relationship with LSM2 expression. **D** Spearman correlation between Expression (exp) of LSM4 and Immunoinhibitors across SKCM. Top 4 immunoinhibitors displaying the greatest Spearman’s correlation with LSM4 expression. **E** Spearman correlation between Expression (exp) of LSM4 and Immunostimulators across SKCM. Top 4 immunostimulators displaying the greatest correlation with LSM4 expression. **F** Spearman correlation between Expression (exp) of LSM4 and MHC across SKCM. Top 4 MHC molecules displaying the greatest relationship with LSM4 expression

Based on the above analysis, it was demonstrated that LSM2 and LSM4 are associated with poor prognosis and have a significant relationship with immune cells. To further explore the regulatory effect of LSM2 and LSM4 on immune molecules in SKCM, the TISIDB database was used to perform an integrated analysis to predict the correlation between LSM expression and immunomodulators. Immunomodulators could be further classified into immunoinhibitors, immunostimulators, and major histocompatibility complex (MHC) molecules. The correlation between LSM2 expression and immunoinhibitors is shown in Fig. 11A, and the expression of LSM2 has the strongest correlation with CD274 (Spearman: ρ = −0.267, *p* = 4.11e−09), PDCD1LG2 (Spearman: ρ = −0.267, *p* = 4.11e−09), TGFBR1 (Spearman: ρ = −0.242, *p* = 1.1e−07), and CSF1R (Spearman: ρ = −0.216, *p* = 2.26e−06). We also compared the correlation between LSM2 expression and immunostimulators (Fig. 11B). The immunostimulators displaying the greatest correlation included TNFSF4 (Spearman: ρ = −0.315, *p* = 3.35e−12), TNFSF13B (Spearman: ρ = −0.311, *p* = 6.07e−12), IL2RA (Spearman: ρ = −0.305, *p* = 1.74e−11), and TNFSF14 (Spearman: ρ = −0.273, *p* = 1.94e−09). Figure 11C presents the correlation between LSM2 expression and MHC molecules, and the MHC molecules displaying the greatest correlation included TAPBP (Spearman: ρ = 0.248, *p* = 5.26e−08), B2M (Spearman: ρ = −0.239, *p* = 1.7e−07), HLA-A (Spearman: ρ = 0.21, *p* = 4.61e−06), and HLA-DOA (Spearman: ρ = −0.199, *p* = 1.33e−05).

The correlation between LSM4 expression and immunoinhibitors is shown in Fig. 11D, and the expression of LSM4 had the strongest correlation with PDCD1LG2 (Spearman: ρ = −0.305, *p* = 1.61e−11), CD274 (Spearman: ρ = −0.287, *p* = 2.59e−10), KDR (Spearman: ρ = −0.267, *p* = 4.31e−09), and TGFBR1 (Spearman: ρ = −0.258, *p* = 1.54e−08). We also compared the correlation between LSM4 expression and immunostimulators (Fig. 11E). LSM4 was significantly correlated with immunostimulators, such as TNFRSF14 (Spearman: ρ = 0.412, *p* < 2.2e−16), CD28 (Spearman: ρ = −0.311, *p* = 6.17e−12), IL2RA (Spearman: ρ = −0.301, *p* = 3.24e−11), and TNFSF13B (Spearman: ρ = −0.298, *p* = 4.85e−11). LSM4 expression was also associated with MHC molecules (Fig. 11F), including HLA-DOA (Spearman: ρ = −0.268, *p* = 4.03e−09), HLA-DMB (Spearman: ρ = −0.226, *p* = 7.27e−07), B2M (Spearman: ρ = −0.2, *p* = 1.24e−05), and HLA-DRA (Spearman: ρ = −0.178, *p* = 0.000105). These results suggest that LSM2 and LSM4 affect the prognosis of patients and may play a role as immune regulators in SKCM.

### Comprehensive results of LSM2

#### Expression pattern and prognostic value of LSM2

Pan-cancer analysis indicated the aberrant expression of LSM2 in human cancers (Additional file 2: Figure S2). The results showed that LSM2 expression was significantly increased in 10 tumors, including diffuse large B-cell lymphoma (DLBC), glioblastoma multiforme (GBM), lower-grade glioma (LGG), liver hepatocellular carcinoma (LIHC), lung squamous cell carcinoma (LUSC), ovarian cancer (OV), pancreatic adenocarcinoma (PAAD), SKCM, testicular germ cell tumors (TGCT), and thymoma (THYM). Meanwhile, LSM2 expression was decreased in kidney chromophobe (KICH) and acute myeloid leukemia (LAML). To explore the effect of LSM2 expression on the prognosis of 33 types of cancer, univariate Cox regression analysis indicated that LSM2 overexpression was inversely correlated with poor OS in SKCM (*p* = 0.013), LIHC (*p* = 0.005), kidney renal clear cell carcinoma (KIRC) (*p* = 0.006), kidney renal papillary cell carcinoma (KIRP) (*p* = 0.005), OV (*p* = 0.024), rectum adenocarcinoma (READ) (*p* = 0.017), sarcoma (SARC) (*p* = 0.017), TGCT (*p* = 0.017), THYM (*p* = 0.013), uveal melanoma (UVM) (*p* = 0.003), and lung adenocarcinoma (*p* < 0.001). Therefore, LSM2 overexpression in SKCM, LIHC, OV, TGCT, and THYM had similar negative effects on the prognosis. Detailed results are shown in forest charts (Additional file 3: Figure S3).

**Fig. 12:**
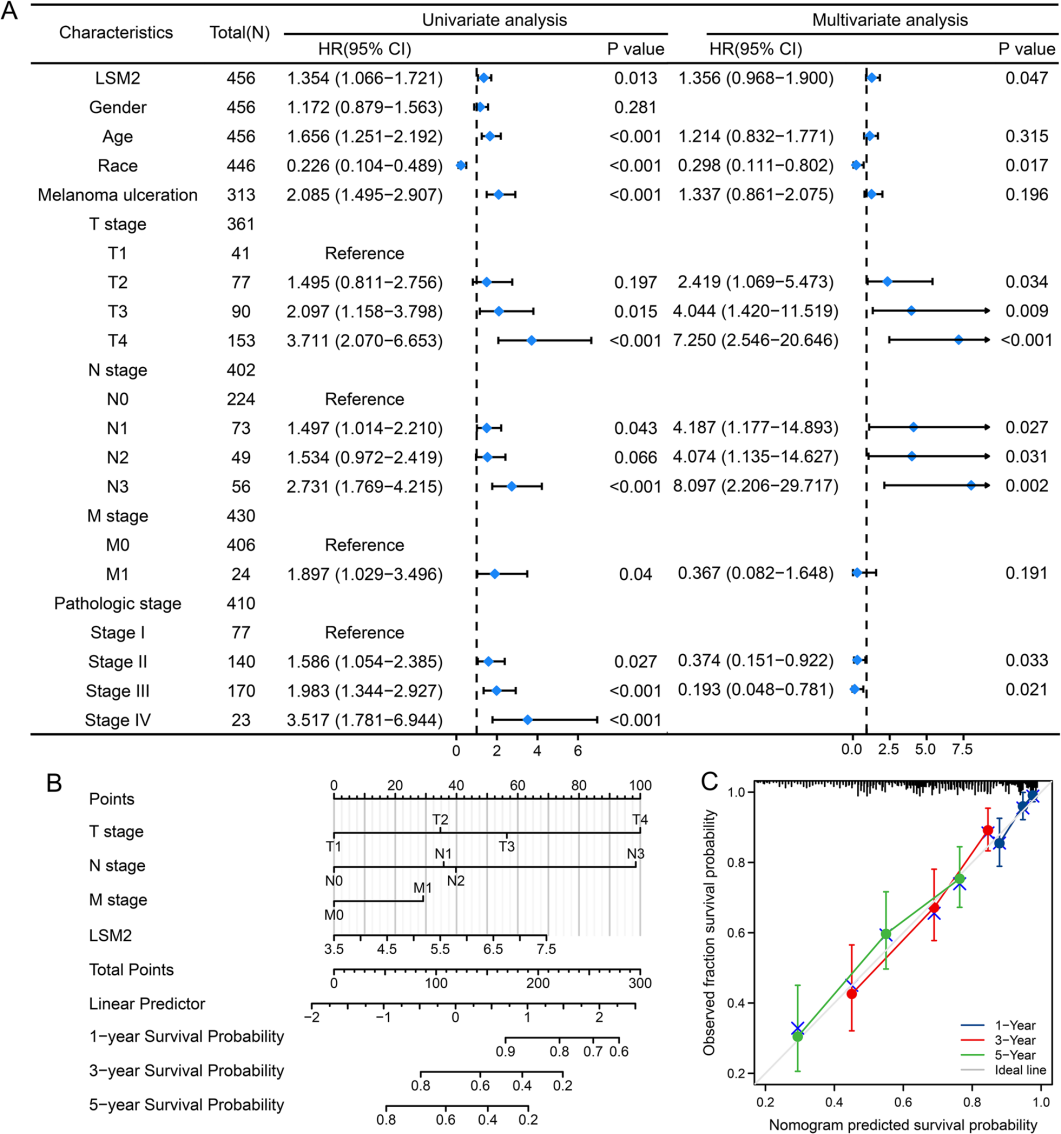
The Cox regression analysis of LSM2 in SKCM and the nomogram model established based on the expression of LSM2 in SKCM. A. The results of univariate and multivariate Cox regression analyses of clinical characteristics and LSM2 expression in the OS of patients with SKCM are represented by forest plots. B. A nomogram based on LSM2 expression for predicting the probability of 1-, 3-, and 5-year OS in patients with SKCM. C. The efficiency of nomograms for OS was validated using calibration plots

Cox regression analysis was conducted to further investigate the role of LSM2 in SKCM (Fig. 12 A). In the univariate analysis, age, race, melanoma ulceration, TNM stage, pathologic stage, and LSM2 expression were significantly related to the prognosis of SKCM in the TCGA database. In the multivariate analysis, race, T stage, N stage, pathologic stage II, pathologic stage III, and LSM2 expression were independent prognostic factors. Incomplete clinical information or the limited number of samples might have resulted in the above differences. To explore the prognostic ability of the above factors, we constructed a nomogram model for 1-, 3-, and 5-year OS prediction (Fig. 12B). The calibration curve displayed a desirable prediction of the nomograms for 1-, 3-, and 5-year clinical outcomes (Fig. 12C). Combined with the above data, LSM2 could be a useful factor for predicting prognosis in SKCM.

**Fig. 13:**
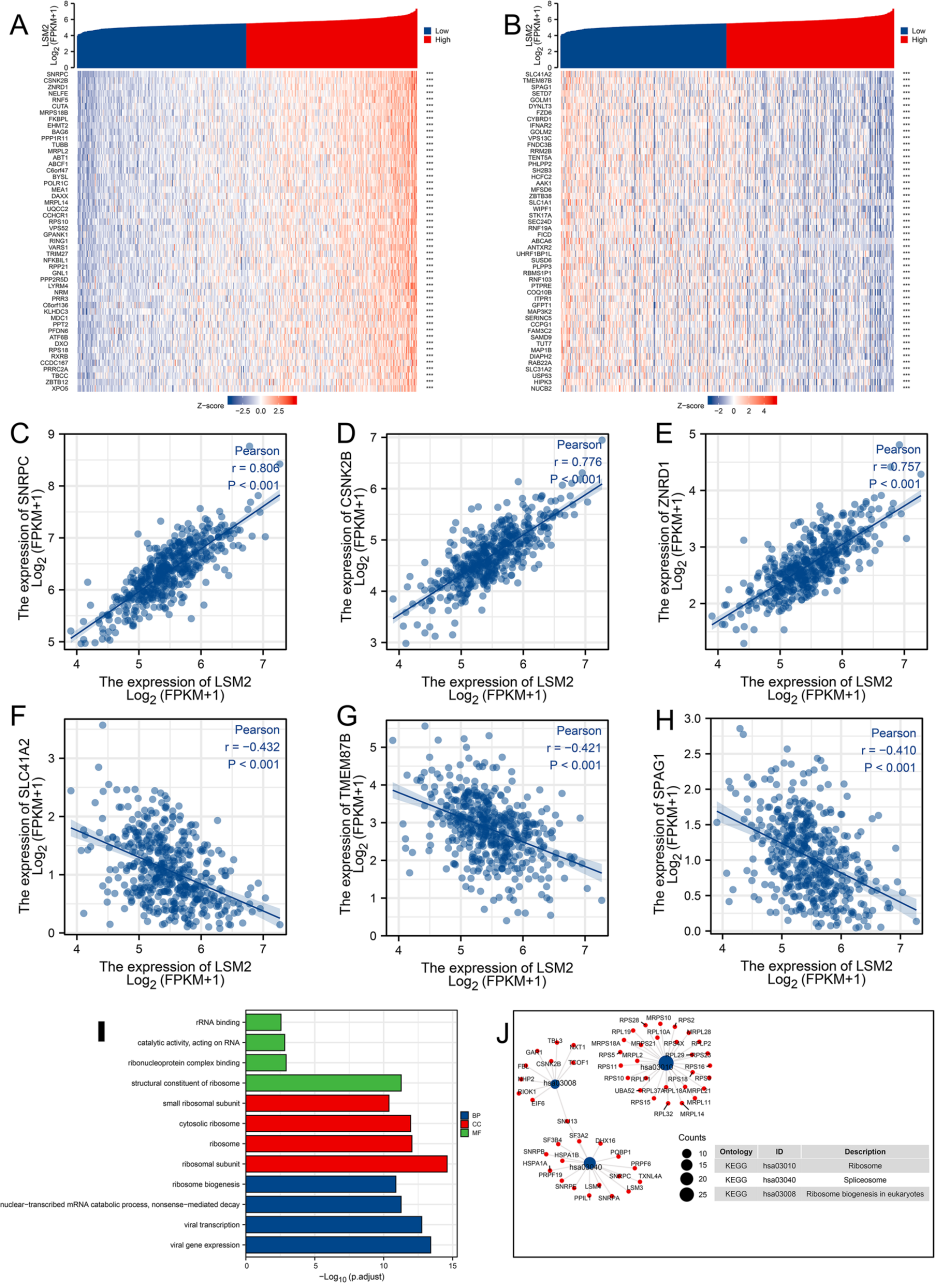
LSM2 co-expression genes and functional enrichment analysis in SKCM. **A** and **B** Heatmap of the top 50 genes that were positively and negatively related to LSM2. **C**–**E**. The top three positively co-expressed genes in the LSM2. **F**–**H**. The top three negatively co-expressed genes in the LSM2. **I** Top four GO terms enriched in BP, CC, and MF. **J** KEGG enrichment of LSM2 co-expression genes, including ribosomes, spliceosomes, and ribosome biogenesis in eukaryotes. The genes involved in each pathway are displayed in the network

#### Functional enrichment analysis of LSM2 in SKCM

Figure 13A and B displayed the top 50 genes positively and negatively associated with LSM2, respectively. LSM2 was positively most co-expressed with SNRPC (R = 0.806, *p* = 7.5226E-109), CSNK2B (R = 0.776, *p* = 9.592E−96), and ZNRD1 (R = 0.757, *p* = 7.861E−89) (Fig. 13C–E), and negatively most co-expressed with SLC41A2 (R = −0.432, *p* = 8.435E−23), TMEM87B (R = −0.421, *p* = 1.256E−21), and SPAG1 (R = −0.410, *p* = 1.538E−20) (Fig. 13F, G, H). LSM2 and co-expression genes were enriched in 71 terms of GO BP, including viral gene expression, viral transcription, nuclear-transcribed mRNA catabolic process, nonsense-mediated decay, and nuclear-transcribed mRNA catabolic process, etc. LSM2 and co-expression genes were enriched in 63 terms of GO CC, including ribosomal subunit, ribosome, cytosolic ribosome, and small ribosomal subunit, etc. There were 19 terms of GO MF of those genes involved in, including structural constituent of ribosome, ribonucleoprotein complex binding, catalytic activity, acting on RNA, and rRNA binding (Fig. 13I). The KEGG [35] analysis displayed that LSM2, and related genes were significantly enriched in ribosome, spliceosome, and ribosome biogenesis in eukaryotes (Fig. 13J)

## Discussion

SKCM is the most aggressive type of skin cancer. Although SKCM represents a minority of all types of skin cancers, it causes the majority of skin cancer-related deaths worldwide [2, 37, 38]. These data and the increasing number of cases make it imperative to identify more specific and sensitive prognostic biomarkers of SKCM. According to previous studies, the LSM family genes were involved in the various types of cancer [26, 31, 32]. We hypothesized that LSM family members could serve as novel biomarkers for SKCM. However, no study has provided an overview of the entire LSM family in SKCM. To our knowledge, this study is the first to comprehensively explore the expression profile of LSM family members and to elucidate the relationships between LSM family members and the prognosis of patients with SKCM.

In this study, we found that LSM2, LSM4, LSM8, LSM10, and LSM12 were highly expressed in SKCM tissues compared with normal skin tissues, whereas LSM1, LSM3, LSM5, LSM7, LSM11, LSM14A, and LSM14B were not significantly upregulated or downregulated. Consistently, another study revealed that LSM2 was overexpressed in basal-like primary tumors [39]. Upregulated LSM4 expression promotes the growth and metastasis of HCC cells by regulating the cell cycle and focal adhesion pathways [33]. In addition, LSM4 overexpression has been associated with rapid cancer progression in BC [31, 40]. Recently, LSM12 was found to be overexpressed in colorectal cancer (CRC) and to play an important role in cancer metastasis and progression [41]. LSM1 downregulation is involved in prostate cancer progression [27], whereas amplification of the LSM1 gene in luminal BC is significantly related to poor clinical outcome [42] and LSM1 expression was significantly related to tumor stage in BC [31]. LSM1 is overexpressed in 87% of human pancreatic tumor samples, and LSM1 knockout has been shown to have therapeutic efficacy in mouse pancreatic cancer models [26]. LSM3 expression was correlated with progression-free survival and strongly associated with the metastatic phenotype of cervical carcinoma [43]. LSM7 overexpression is highly accurate in the preoperative diagnosis of thyroid nodules [44]. These studies displayed a reverse comparison with our expression profile analysis of SKCM. This inconsistency may be due to tumor heterogeneity. Therefore, further in vitro and in vivo studies are required. The different expression characteristics of LSM family genes in cancers can be explained by factors such as genetic alterations, epigenetic changes, and tumor microenvironment (TME) [45–47]. In particular, RNA splicing, transcription, post-translational modification of proteins, and other interactions can lead to different phenotypes [48–50].

Furthermore, in the HPA dataset, LSM2, LSM4, and LSM12 proteins were confirmed to be overexpressed in SKCM tissues compared with normal skin tissues. There are no data on LSM1 and LSM10 expression in the HPA dataset. However, in lung cancers and mesotheliomas, LSM1 protein was overexpressed compared to adjacent normal lung tissue, as measured by immunohistochemistry and western blot analysis [51]. This indicates that the results of the dataset analysis need to be further validated by in vivo and in vitro experiments. In addition, we demonstrated that upregulated expression of LSM2 and LSM4 was significantly associated with shorter OS, whereas LSM6 overexpression was associated with longer OS. Additionally, univariate Cox regression analysis revealed that LSM2, LSM4, and LSM6 expression levels were risk factors influencing the prognosis of SKCM. Multivariate analysis concluded that LSM2 expression and TNM stage were related to adverse clinical outcomes in patients with SKCM. Moreover, a prognostic nomogram model based on TNM stage and LSM2 expression could increase the accuracy of identifying high-risk patients. Moreover, LSM2 and LSM4 showed relatively high accuracy (AUC > 0.8) in predicting the prognosis of patients with SKCM. LSM2 was also an independent predictor of poor prognosis in other tumors [28]. LSM2 overexpression in LIHC, OV TGCT, and THYM had similar negative effects on prognosis. The aberrant DNA methylation of LSM2 in the OV might lead to increased LSM2 expression, thereby promoting tumor development [28]. In terms of DFS, only LSM2 overexpression was associated with DFS in patients with SKCM. In addition, high expression of LSM2 was markedly related to high tumor stage. Another study also confirmed a significant relationship between upregulated LSM2 expression and advanced tumor stage [31].

To explore the cause of the aberrant expression of LSM family genes in SKCM, we further analyzed the genetic variations, GO enrichment, and KEGG annotation of LSM genes in SKCM. These results confirmed that LSM family genes were altered. The amplification and mutation of these genes might be one of the reasons for their aberrant expression. This was also confirmed in another study [52]. Previous studies have revealed that LSM proteins are RBPs and play important roles in RNA metabolism [53]. Sm protein binding is essential for the biogenesis and stability of small nuclear ribonucleoproteins (snRNPs). In this study, GO analysis also revealed that the LSM family and related genes were RBPs and were mainly involved in RNA binding and splicing. KEGG analysis of these genes revealed spliceosome and RNA degradation. Our results also confirmed that LSM genes were most closely related to some snRNPs, such as SNRPD2, SNRPD3, SNRPF, and SNRPE. The copy number variation of SNRPD2 was significantly associated with gene expression and poor prognosis in patients with HCC [54]. SNRPD2 and SNRPD3 were identified as splicing factors and were also critical for various proliferations in BC patients by regulating effective sororin splicing [55]. An RBP-based prognostic prediction model for LUAD also indicated that SNRPE participates in the occurrence and development of cancer [56]. In addition, SNRPE was found to be involved in cell proliferation and the progression of prostate cancer by regulating androgen receptor mRNA expression in cells [57]. SNRPD2 and SNRPF are the core proteins related to below-background radiation, and further affect the proliferation of well-differentiated laryngeal squamous cell carcinoma cells [58]. In addition, some LSM genes can influence circadian rhythms by regulating RNA processing in mammals and plants [59]. LSM3 was identified as a major pathogenic gene of AD, which involved RNA silencing and degradation [29]. Increasing evidence has shown that post-transcriptional regulation plays a critical role in gene expression. RNA splicing is efficiently and precisely conducted by the spliceosome. However, in many cases, the splicing process is flexible enough to produce alternative transcripts that affect protein transcript levels and diversity [60]. Given the importance of RNA splicing, alterations in this process have been implicated in several human cancers. Mutations in some RNA splicing factors, such as SF3B1, SRSF2, and U2AF1, have been found to be involved in the pathogenesis of cancers, such as lung cancer, breast cancer, and pancreatic cancers [61–63]. Tumor cells often take advantage of mutations in RNA splicing to promote growth and invasion [64]. BRAF, NRAS, CDKN2A, and TP53 are known to be significantly mutated in cutaneous melanoma. Mutations in SF3B1 often occur in mucosal melanoma [65]. Recently, one study confirmed that SF3B1 mutations occur in SKCM [66]. Another study also demonstrated that mutations in RBP, including LSM genes, were correlated with splicing aberrations in SKCM [52]. Herein, we discovered that LSM genes in SKCM mainly participate in RNA splicing and degradation. But whether the different alterations of LSM genes influences RNA expression and RNA splicing in SKCM patients and their mechanism of action remain elusive.

In particular, LSM2 had the highest alteration rate (3%) among the LSM family. Altered LSM2 expression was significantly associated with shorter OS in patients with SKCM. Similarly, genetic variants of LSM2 are associated with lung cancer risk [67]. Our findings also suggest that LSM2 is an independent risk factor for poor LUAD prognosis. Functional enrichment analysis further confirmed that LSM2 mainly participated in viral gene expression and RNA metabolism in eukaryotes, similar to the LSM family. We found that the top three genes co-expressed with LSM2 were SNRPC, CSNK2B, and ZNRD1. Gene expression data revealed that CSNK2B was highly expressed in CRC. Moreover, in vitro and in vivo experiments indicated that upregulated CSNK2B promotes CRC cell proliferation [68]. Another study confirmed that SNRPC is involved in the regulation of spliceosomes, ribosomes, and proteasome signaling. SNRPC promotes HCC cell motility [69]. Overexpression of ZNRD1 can promote the multidrug-resistant phenotype of GC cells via upregulation of P-glycoprotein [70, 71]. Therefore, we hypothesized that LSM2 and its co-expressed genes may play a synergistic role in promoting the initiation and development of SKCM.

Moreover, the occurrence and progression of tumors are not only related to gene mutations, but also involve complex interactions between malignant cells and TME. Previous studies have indicated that the immune infiltration of NK CD56 bright cells significantly affects the survival of SKCM [72]. This finding is consistent with the results of the present study. High expression of LSM2 and LSM4 was positively related to immune infiltration of NK CD56 bright cells and significantly affected prognosis. Tcm has excellent anti-tumor ability [73]. Tcm and its derived clonal T cells are highly effective anti-tumor immune T cells [74]. Tcm can be activated by tumor antigens to directly kill tumor [75]. In a melanoma patient, Tcm cells were found to have the ability to self-renew and replicate, and survive in vivo for a long time to achieve persistence of anti-tumor effects [76]. In this study, Tcm infiltration was positively correlated with LSM8 and LSM12 but negatively correlated with LSM2 and LSM4, which was consistent with the effect of genes on tumor prognosis.

Immune checkpoint inhibitors (ICIs) are an important new strategy for tumor immunotherapy, and have gradually improved the prognosis of patients with a variety of cancers [77–79]. In addition, we analyzed the associations between LSMs (LSM2 and LSM4) and the expression of immunoinhibitors, immunostimulators, and MHC molecules in various types of human cancers. Interestingly, we found that both LSM2 and LSM4 had significant associations with CD274, PDCD1LG2, TGFBR1, TNFSF14, IL2RA, HLA-DOA, and B2M in patients with SKCM. TGFBR1 [80], PDCD1LG2 [79], CD274 [81], and TNFSF14 [80] are involved in tumor immune evasion and are effective targets for tumor immunotherapy. Therefore, this study provides new insights into the roles of LSM genes in immune cell infiltration and their potential as immunotherapy targets in SKCM.

This study has some limitations. First, the possible expression profile and functions of LSM2 have not yet been validated in vivo or in vitro. Further research on the function of LSM2 in clinical and basic experiments is required. Second, this study preliminarily explored the expression profile, prognostic value, and biological function of LSM family genes, especially LSM2, in SKCM. Bioinformatics analysis of genes using different databases or analysis tools might result in different results. Therefore, it is necessary to validate the LSM genes in clinical samples and cell lines.

## Conclusions

In summary, diverse associations between LSM family genes and SKCM were identified for the first time. Our findings revealed that LSM family members were differentially expressed in patients with SKCM. Furthermore, the LSM family played important roles in RNA metabolism. Upregulated LSM2 is significantly associated with shorter OS and DFS in patients with SKCM, which may be caused by abnormal RNA metabolism, LSM2 alteration or the regulation of immune check point genes. Therefore, LSM2 might be a potential poor prognostic indicator and immunotherapy target for SKCM.

## Supplementary Information

**Additional file 1**.** Supplementary Fig S1**. The expression of LSM family members in SKCM was analyzed using univariate Cox regression as a predictor of OS. The results are presented as forest plots. LSM2, LSM4, and LSM6 significantly influenced the OS of patients with SKCM.

**Additional file 2**.** Supplementary Fig S2**. Expression profile of LSM2 in pan-cancer. Red indicates significant upregulation, while green indicates downregulation. Black indicates no significant differences.

**Additional file 3**.** Supplementary Fig S3**. The forest plot indicates the results of OS analysis of LSM2 in pan-cancers by univariate Cox regression.

**Additional file 4**.** Supplementary Table S1**. The correlation coefficients of the LSMS family genes in SKCM were explored using Spearman’s test.

**Additional file 5**.** Supplementary Table S2**. The relationship between LSM family genes in SKCM was investigated using Spearman’s test. The p-values are presented in this table.

## Data Availability

The datasets generated during the study are available in the TCGAGDC (https://portal.gdc.cancer.gov/).

## Abbreviations

AD: Alzheimer’s disease
AUC: Area under the curve
aDC: Activated dendric cells
BC: Breast cancer
BP: Biological process
cBioPortal: CBio Cancer Genomics Portal
CC: Cellular component
CI: Confidence interval
CRC: Colorectal cancer
DC: Dendritic cell
DEGs: Differentially expressed genes
DFS: Disease-free survival
DLBC: Diffuse large B-cell lymphoma
GDC: Genomic Data Commons
GEPIA: Gene Expression Profiling Interactive Analysis
GO: Gene Ontology
GTEx: Genotype Tissue Expression
GBM: Glioblastoma multiforme
HCC: Hepatocellular carcinoma
HPA: Human Protein Atlas
HR: Hazard ratio
iDC: Immature DC
KEGG: Kyoto Encyclopedia of Genes and Genomes
KICH: Kidney chromophobe
KIRC: Kidney renal clear cell carcinoma
KIRP: Kidney renal papillary cell carcinoma
LAML: Acute myeloid leukemia
LSM: Like-Smith
LGG: Lower-grade glioma
LIHC: Liver hepatocellular carcinoma
LUSC: Lung squamous cell carcinoma
MHC: Major histocompatibility complex
MF: Molecular function
NK cells: Natural killer cells
OS: Overall survival
OV: Ovarian cancer
PAAD: Pancreatic adenocarcinoma
PDAC: Pancreatic ductal adenocarcinoma
pDC: Plasmacytoid DC
RBP: RNA binding protein
ROC: Receiver operating characteristic
READ: Rectum adenocarcinoma
SKCM: Skin cutaneous melanoma
snRNA: Small nuclear RNA
SARC: Sarcoma
snRNPs: Small nuclear ribonucleoproteins
TCGA: The Cancer Genome Atlas
Tcm: T central memory
Th2 cells: Type 2 Th cells
Tgd: T gamma delta
TFH: T follicular helper
Treg: Regulatory T cell
TGCT: Testicular germ cell tumors
THYM: Thymoma
TME: Tumor microenvironment
UVM: Uveal melanoma
