# Supplementary material for: Expression profile and prognostic values of LSM family in skin cutaneous melanoma
Source: BMC Med Genomics. 2022 Nov 12;15:238. doi: 10.1186/s12920-022-01395-6 (PMC9656080; doi:10.1186/s12920-022-01395-6)
Supplement: Supplementary file 1 — Additional file 1. Supplementary Fig S1. The expression of LSM family members in SKCM was analyzed using univariate Cox regression as a predictor of OS. The results are presented as forest plots. LSM2, LSM4, and LSM6 significantly influenced the OS of patients with SKCM. [file 12920_2022_1395_MOESM1_ESM.docx]

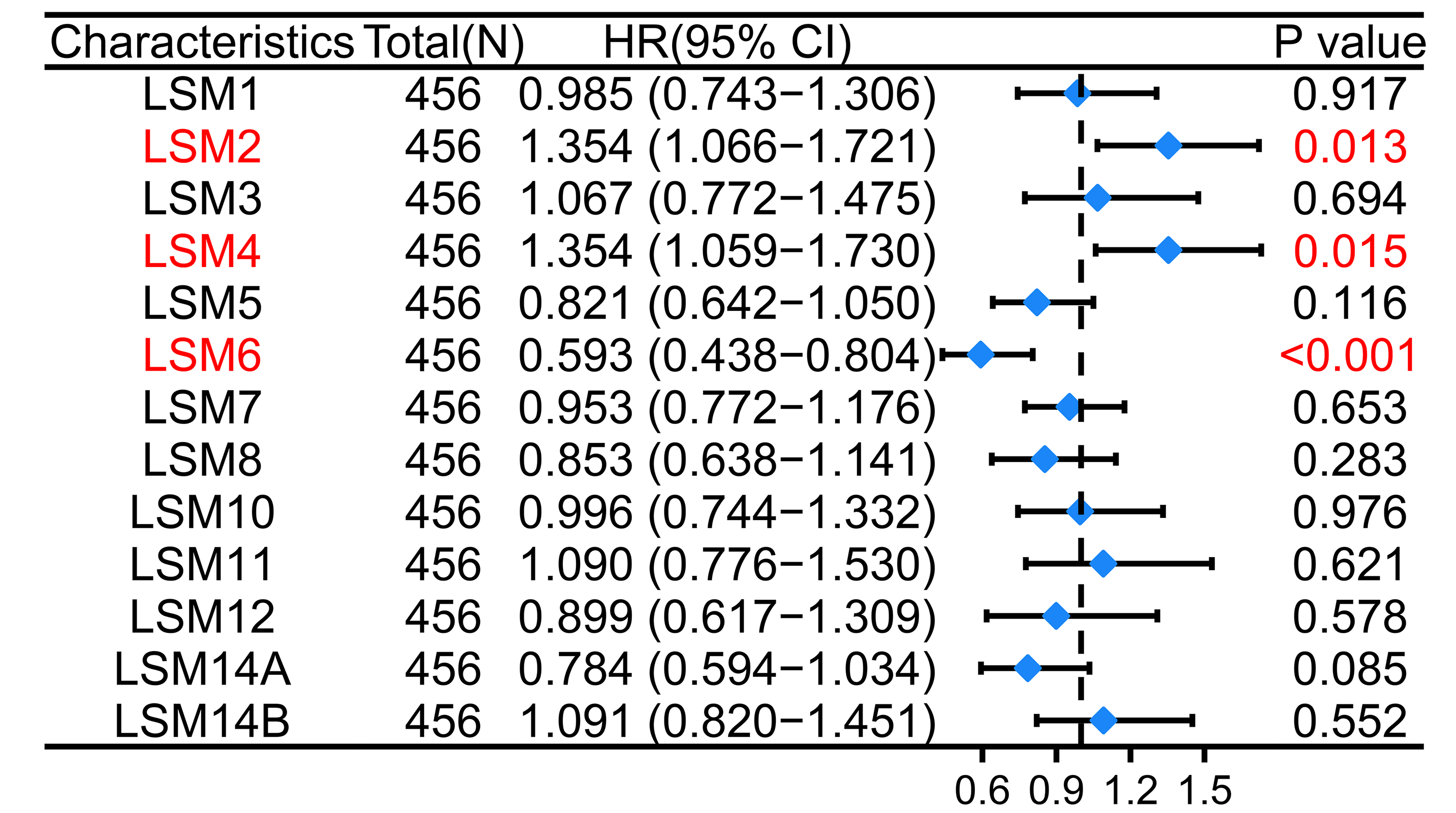


**Supplementary Fig S1** The expression of LSM family members in SKCM was analyzed using univariate Cox regression as a predictor of OS. The results are presented as forest plots. LSM2, LSM4, and LSM6 significantly influenced the OS of patients with SKCM.
