# Supplementary material for: Expression profile and prognostic values of LSM family in skin cutaneous melanoma
Source: BMC Med Genomics. 2022 Nov 12;15:238. doi: 10.1186/s12920-022-01395-6 (PMC9656080; doi:10.1186/s12920-022-01395-6)
Supplement: Supplementary file 2 — Additional file 2. Supplementary Fig S2. Expression profile of LSM2 in pan-cancer. Red indicates significant upregulation, while green indicates downregulation. Black indicates no significant differences. [file 12920_2022_1395_MOESM2_ESM.docx]

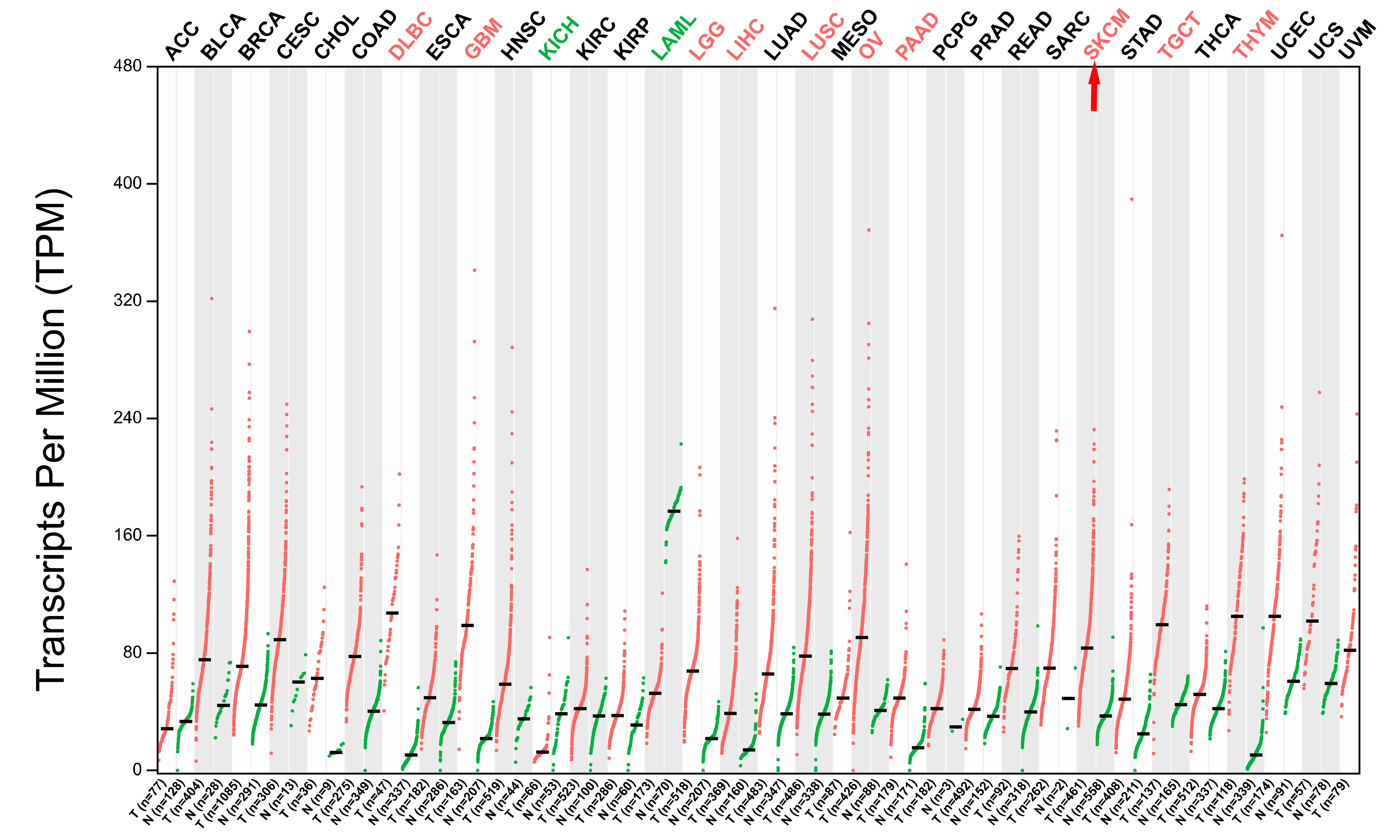


**Supplementary Fig S2** Expression profile of LSM2 in pan-cancer. Red indicates significant upregulation, while green indicates downregulation. Black indicates no significant differences.
