# Supplementary material for: Expression profile and prognostic values of LSM family in skin cutaneous melanoma
Source: BMC Med Genomics. 2022 Nov 12;15:238. doi: 10.1186/s12920-022-01395-6 (PMC9656080; doi:10.1186/s12920-022-01395-6)
Supplement: Supplementary file 3 — Additional file 3. Supplementary Fig S3. The forest plot indicates the results of OS analysis of LSM2 in pan-cancers by univariate Cox regression. [file 12920_2022_1395_MOESM3_ESM.docx]

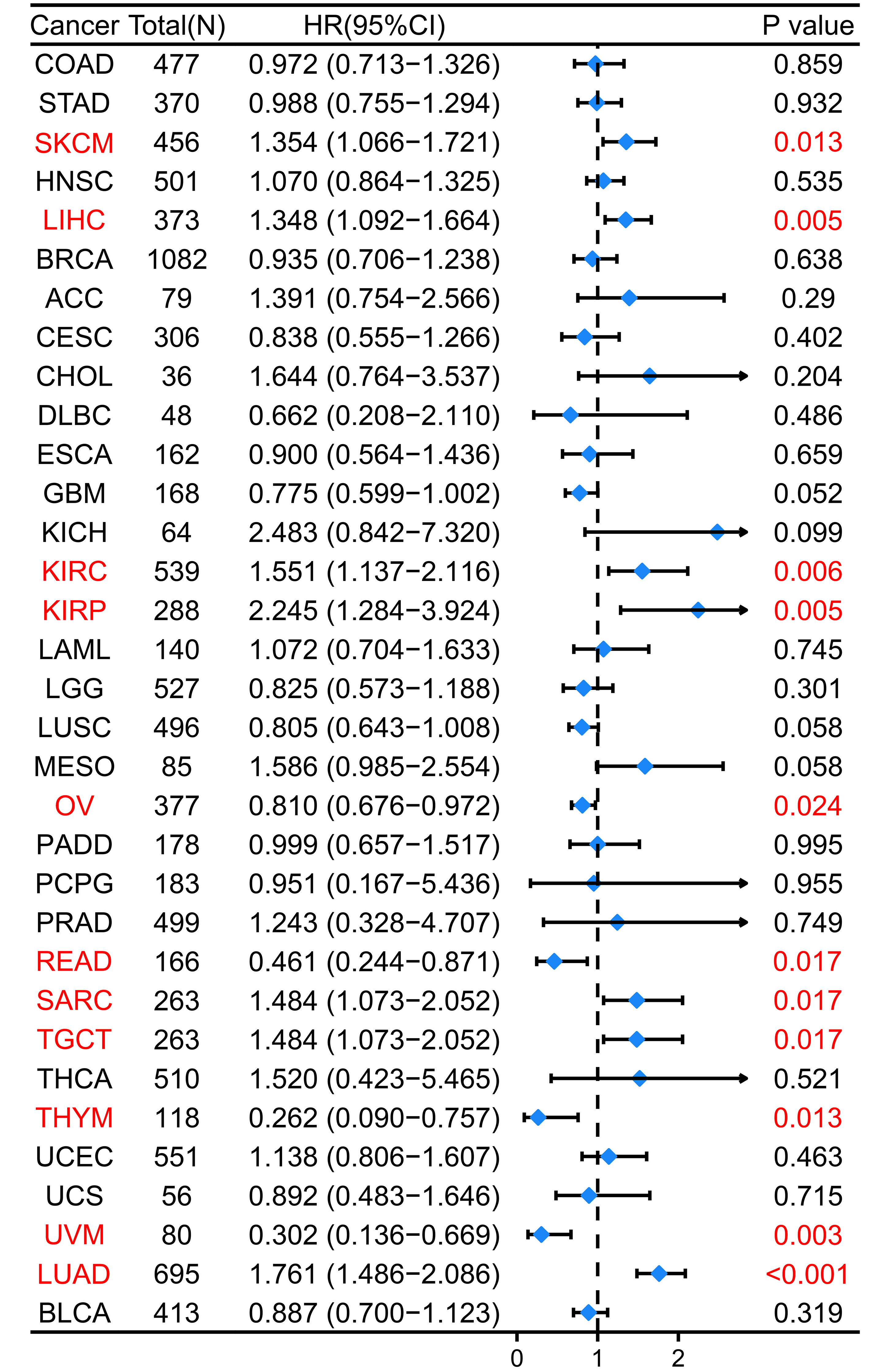


**Supplementary Fig S3** The forest plot indicates the results of OS analysis of LSM2 in pan-cancers by univariate Cox regression.
