# Supplementary material for: Expression profile and prognostic values of LSM family in skin cutaneous melanoma
Source: BMC Med Genomics. 2022 Nov 12;15:238. doi: 10.1186/s12920-022-01395-6 (PMC9656080; doi:10.1186/s12920-022-01395-6)
Supplement: Supplementary file 4 — Additional file 4. Supplementary Table S1. The correlation coefficients of the LSMS family genes in SKCM were explored using Spearman’s test. [file 12920_2022_1395_MOESM4_ESM.docx]

|  | **LSM1** | **LSM2** | **LSM3** | **LSM4** | **LSM5** | **LSM6** | **LSM7** | **LSM8** | **LSM10** | **LSM11** | **LSM12** | **LSM14A** | **LSM14B** |
| --- | --- | --- | --- | --- | --- | --- | --- | --- | --- | --- | --- | --- | --- |
| **LSM1** | 1 | 0.03 | 0.24 | 0.07 | 0.31 | 0.3 | 0.15 | 0.32 | 0.09 | 0.12 | 0.22 | 0.16 | -0.01 |
| **LSM2** | 0.03 | 1 | 0.31 | 0.33 | 0.11 | 0.06 | 0.19 | 0.04 | 0.08 | 0.03 | -0.04 | 0.03 | 0.16 |
| **LSM3** | 0.24 | 0.31 | 1 | 0.25 | 0.42 | 0.19 | 0.17 | 0.31 | 0.2 | 0.08 | 0.09 | 0.08 | 0.18 |
| **LSM4** | 0.07 | 0.33 | 0.25 | 1 | 0.13 | -0.07 | 0.47 | 0.17 | 0.41 | -0.24 | -0.11 | -0.14 | 0.09 |
| **LSM5** | 0.31 | 0.11 | 0.42 | 0.13 | 1 | 0.46 | 0.34 | 0.57 | 0.07 | 0.16 | 0.12 | 0.26 | 0.11 |
| **LSM6** | 0.3 | 0.06 | 0.19 | -0.07 | 0.46 | 1 | 0.21 | 0.35 | -0.04 | 0.26 | 0.17 | 0.41 | 0.09 |
| **LSM7** | 0.15 | 0.19 | 0.17 | 0.47 | 0.34 | 0.21 | 1 | 0.36 | 0.2 | -0.06 | -0.22 | 0.08 | 0.03 |
| **LSM8** | 0.32 | 0.04 | 0.31 | 0.17 | 0.57 | 0.35 | 0.36 | 1 | 0.11 | 0.05 | -0.02 | 0.07 | 0.09 |
| **LSM10** | 0.09 | 0.08 | 0.2 | 0.41 | 0.07 | -0.04 | 0.2 | 0.11 | 1 | -0.4 | -0.02 | -0.32 | 0.03 |
| **LSM11** | 0.12 | 0.03 | 0.08 | -0.24 | 0.16 | 0.26 | -0.06 | 0.05 | -0.4 | 1 | 0.28 | 0.53 | 0.12 |
| **LSM12** | 0.22 | -0.04 | 0.09 | -0.11 | 0.12 | 0.17 | -0.22 | -0.02 | -0.02 | 0.28 | 1 | 0.31 | 0.21 |
| **LSM14A** | 0.16 | 0.03 | 0.08 | -0.14 | 0.26 | 0.41 | 0.08 | 0.07 | -0.32 | 0.53 | 0.31 | 1 | 0.14 |
| **LSM14B** | -0.01 | 0.16 | 0.18 | 0.09 | 0.11 | 0.09 | 0.03 | 0.09 | 0.03 | 0.12 | 0.21 | 0.14 | 1 |

Table S1 The correlation coefficients of the LSMS family genes in SKCM were explored using Spearman’s test.
