# Supplementary material for: Expression profile and prognostic values of LSM family in skin cutaneous melanoma
Source: BMC Med Genomics. 2022 Nov 12;15:238. doi: 10.1186/s12920-022-01395-6 (PMC9656080; doi:10.1186/s12920-022-01395-6)
Supplement: Supplementary file 5 — Additional file 5. Supplementary Table S2. The relationship between LSM family genes in SKCM was investigated using Spearman’s test. The p-values are presented in this table. [file 12920_2022_1395_MOESM5_ESM.docx]

| LSM1 | LSM2 | LSM3 | LSM4 | LSM5 | LSM6 | LSM7 | LSM8 | LSM10 | LSM11 | LSM12 | LSM14A | LSM14B |  |
| --- | --- | --- | --- | --- | --- | --- | --- | --- | --- | --- | --- | --- | --- |
| LSM1 | NA | 0.48 | 0 | 0.11 | 0 | 0 | 0 | 0 | 0.05 | 0.01 | 0 | 0 | 0.88 |
| LSM2 | 0.48 | NA | 0 | 0 | 0.01 | 0.19 | 0 | 0.42 | 0.09 | 0.47 | 0.38 | 0.57 | 0 |
| LSM3 | 0 | 0 | NA | 0 | 0 | 0 | 0 | 0 | 0 | 0.07 | 0.05 | 0.1 | 0 |
| LSM4 | 0.11 | 0 | 0 | NA | 0 | 0.13 | 0 | 0 | 0 | 0 | 0.02 | 0 | 0.04 |
| LSM5 | 0 | 0.01 | 0 | 0 | NA | 0 | 0 | 0 | 0.11 | 0 | 0.01 | 0 | 0.02 |
| LSM6 | 0 | 0.19 | 0 | 0.13 | 0 | NA | 0 | 0 | 0.37 | 0 | 0 | 0 | 0.06 |
| LSM7 | 0 | 0 | 0 | 0 | 0 | 0 | NA | 0 | 0 | 0.21 | 0 | 0.08 | 0.58 |
| LSM8 | 0 | 0.42 | 0 | 0 | 0 | 0 | 0 | NA | 0.02 | 0.29 | 0.72 | 0.15 | 0.05 |
| LSM10 | 0.05 | 0.09 | 0 | 0 | 0.11 | 0.37 | 0 | 0.02 | NA | 0 | 0.69 | 0 | 0.59 |
| LSM11 | 0.01 | 0.47 | 0.07 | 0 | 0 | 0 | 0.21 | 0.29 | 0 | NA | 0 | 0 | 0.01 |
| LSM12 | 0 | 0.38 | 0.05 | 0.02 | 0.01 | 0 | 0 | 0.72 | 0.69 | 0 | NA | 0 | 0 |
| LSM14A | 0 | 0.57 | 0.1 | 0 | 0 | 0 | 0.08 | 0.15 | 0 | 0 | 0 | NA | 0 |
| LSM14B | 0.88 | 0 | 0 | 0.04 | 0.02 | 0.06 | 0.58 | 0.05 | 0.59 | 0.01 | 0 | 0 | NA |

Table S2 The relationship between LSM family genes in SKCM was investigated using Spearman’s test. The p-values are presented in this table.
